# Structural Insights Into the Effects of Interactions With Iron and Copper Ions on Ferritin From the Blood Clam *Tegillarca granosa*

**DOI:** 10.3389/fmolb.2022.800008

**Published:** 2022-03-11

**Authors:** Tinghong Ming, Qinqin Jiang, Chunheng Huo, Hengshang Huan, Yan Wu, Chang Su, Xiaoting Qiu, Chenyang Lu, Jun Zhou, Ye Li, Jiaojiao Han, Zhen Zhang, Xiurong Su

**Affiliations:** ^1^ State Key Laboratory for Managing Biotic and Chemical Threats to the Quality and Safety of Agro-products, Ningbo University, Ningbo, China; ^2^ School of Marine Science, Ningbo University, Ningbo, China; ^3^ College of Food and Pharmaceutical Sciences, Ningbo University, Ningbo, China; ^4^ Zhejiang Collaborative Innovation Center for High Value Utilization of Byproducts from Ethylene Project, Ningbo Polytechnic College, Ningbo, China

**Keywords:** blood clam *Tegillarca granosa*, ferritin, crystal structure, Fe^2+^ ion, Cu^2+^ ion, catalytic activity

## Abstract

In addition to its role as an iron storage protein, ferritin can function as a major detoxification component in the innate immune defense, and Cu^2+^ ions can also play crucial antibacterial roles in the blood clam, *Tegillarca granosa*. However, the mechanism of interaction between iron and copper in recombinant *Tegillarca granosa* ferritin (TgFer) remains to be investigated. In this study, we investigated the crystal structure of TgFer and examined the effects of Fe^2+^ and Cu^2+^ ions on the TgFer structure and catalytic activity. The crystal structure revealed that TgFer presented a typically 4–3–2 symmetry in a cage-like, spherical shell composed of 24 identical subunits, featuring highly conserved organization in both the ferroxidase center and the 3-fold channel. Structural and biochemical analyses indicated that the 4-fold channel of TgFer could be serviced as potential binding sites of metal ions. Cu^2+^ ions appear to bind preferentially with the 3-fold channel as well as ferroxidase site over Fe^2+^ ions, possibly inhibiting the ferroxidase activity of TgFer. Our results present a structural and functional characterization of TgFer, providing mechanistic insight into the interactions between TgFer and both Fe^2+^ and Cu^2+^ ions.

## Introduction

Ferritin represents a ubiquitous class of nanocage-like proteins that are primarily involved in maintaining iron homeostasis (Bou-Abdallah et al., 2018). Ferritin is known to be an essential protein that plays dual roles in iron storage and detoxification in almost all living systems (Plays et al., 2021). Although iron is a crucial element for virtually all living species, excess iron is greatly harmful to living cells due to its highly reactive nature (Corsi et al., 2002). Accordingly, ferritin plays a central role in taking up iron (as Fe^2+^) and storing it in a stable and unreactive form (as Fe^3+^-oxide/hydroxide) (Theil, 2012). Estimations suggest that up to 4,000 iron atoms can be stored inside the inner cavity of a single ferritin molecule (Plays et al., 2021). Despite significant differences in the primary sequences of different ferritin proteins, the tertiary and quaternary structures of known ferritins have been demonstrated to be strikingly similar.

Generally, the canonical ferritin structure is composed of 24 subunits. Ferritin can be found in both eukaryotes and prokaryotes, and these highly analogous subunits assemble a large, hollow, symmetrical, spherical shell (Theil, 2012; Plays et al., 2021). A canonical ferritin subunit folds into a four-helix bundle, coupling with a fifth, C-terminal, short-helix E at an approximate angle of 60° (Harrison and Arosio, 1996; Hempstead et al., 1997; Laghaei et al., 2013). The subunits reversibly assemble into a spherically shaped structure with 4–3–2-point symmetries and inner and outer diameters of approximately 8 and 12 nm, respectively (Theil, 2012). The quaternary structure of ferritin contains eight 3-fold channels and six 4-fold channels, with each channel forming from the joining of three and four subunits, respectively. Some small pores with diameters ranging from 0.2 to 0.5 nm and the 3–4 nm in length have been identified along these 3- and 4-fold channels (Harrison and Arosio, 1996; Honarmand Ebrahimi et al., 2015). Although numerous studies have suggested that both the 3-fold and 4-fold channels can act as Fe^2+^ ion entry points into the cage cavity, evidence has only been provided to support ion entry through the highly hydrophilic 3-fold channels in animal ferritin (Lv et al., 2014; Honarmand Ebrahimi et al., 2015; Aronovitz et al., 2016). Among them, mammalian ferritins, apart from mitochondrial and serum ferritin, are predominantly heteropolymers composed of H (heavy, molecular weight of approx. 21 kDa) and L (light, molecular weight of approx. 19 kDa) subunits at variable ratios in the cytosol (Jiang et al., 2020). H-subunit typically contains a di-iron ferroxidase center capable of rapidly oxidizing ferrous (Fe^2+^) ions, whereas the L-subunit lacks such ferroxidase center but does have a nucleation site (Pozzi et al., 2017). Ferritins from lower vertebrate (e.g., bullfrogs and fish) and invertebrate species dispose with a third subunit type, named the Hʹ-subunit, which harbors the residues forming both the ferroxidase center and the nucleation site (Domínguez-Vera et al., 2010; Scudiero et al., 2013; Pozzi et al., 2015b). However, the biological structures and catalytic characteristics of marine invertebrate ferritins and their iron translocation pathways remain relatively unknown.

Previous reports have demonstrated that the hydrophilic 3-fold channel of ferritin can also allow the transport of various other divalent metal ions, e.g., Cu^2+^, Co^2+^, and others, in addition to the uptake of Fe^2+^ ions (Tosha et al., 2010; Maity et al., 2019). Although the binding interactions of distinct ferritins with other metal ions vary considerably, the other ions can have noticeable effects on the structure and function of ferritins (McKnight et al., 1997; Baaghil et al., 2002; Ardini et al., 2018). Notably, previous researches had revealed that these transition metal cations binding in ferritin could selectively inhibit ferritin enzyme activity, mainly by blocking Fe^2+^ ion access, and the order of metal ion–protein binding stabilities was Mn^2+^ << Co^2+^ < Cu^2+^ < Zn^2+^ ion (McKnight et al., 1997; Behera and Theil, 2014). Among them, Cu^2+^ ion has been demonstrated to have an important effect on the capability of iron oxidation at the ferroxidase site in ferritins. Baaghil et al. reported that the presence of Cu^2+^ ion was found to significantly enhance the rate of Fe^2+^ ion oxidation and subsequent core formation (Baaghil et al., 2002). McKnight et al. (1997) found that Cu^2+^ ion could bind tightly to horse spleen apoferritin, and had a catalytic effect on the aerobic oxidative uptake of Fe^2+^ ion. Therefore, despite various biochemical research studies examining the performance of loaded ferritin, the molecular mechanisms that underlie iron or copper uptake and ion interactions with marine invertebrate ferritin remain largely speculative.

The blood clam, *Tegillarca granosa*, is a marine invertebrate with important economic value that is broadly distributed in the sandy muds of the intertidal zone along the east coast of China and Southeast Asia. The bivalve shellfish *T. granosa* belongs to the family Arcidae, in the class Bivalvia (phylum Mollusca), and represents one of the rare invertebrates that possess hemoglobin-containing red hemocytes in the hemolymph (Wang et al., 2021). Both ferritin and Cu^2+^ ions can play crucial roles in the innate immune defense and antibacterial activity of *T. granosa* (Jin et al., 2011; Wang et al., 2016; Wang et al., 2021); however, the interactions between *T. granosa* ferritin (TgFer) and both iron and copper ions remain unclear. In the present study, we present the first crystal structure for recombinant TgFer. In addition, the crystal structures of Cu^2+^-loaded TgFer (TgFer + Cu), Fe^2+^-loaded TgFer (TgFer + Fe), and Fe^2+^-loaded TgFer + Cu (TgFer + CuFe) were successfully determined. Accompanying biochemical experiments examining different ion-loaded TgFer molecules were also performed in this study. The structural and functional characterization reported here provides a comprehensive overview of the molecular mechanisms of TgFer in *T. granosa*.

## Materials and Methods

### Cloning, Expression, and Purification

TgFer cDNA has been reported to be 895 bp in length, consisting of a 163-bp 5′-untranslated region (UTR), a 213-bp 3′-UTR, and a 519-bp complete open reading frame that encodes a 172 amino acid polypeptide (Jin et al., 2011). The TgFer gene was amplified from the gene encoding full-length *T. granosa* ferritin (GenBank: ADC34696.1). PCR products were digested and subcloned into a pET-28a (+) expression vector (Novagen) containing *Nde*I and *Xho*I sites, followed by an N-terminal SUMO tag. The primer sets for TgFer (forward: 5′-CACCAT​ATGTCG​GAC​TCA​GAA​GTC​AAT​C-3′; reverse: 5′- CACCTC​GAGTTA​GCT​GCT​CAT​GGT​TTC​TTT-3′) were designed to provide *Nde*I and *Xho*I sites at the 5 and 3′ termini, respectively. The recombinant plasmid was transformed into *Escherichia coli* BL21 (DE3) cells (Novagen). When the cells reached an optical density at 600 nm (OD_600nm_) ranging from 0.4 to 0.6, they were induced with a final concentration of 0.5 mM isopropyl β-d-1-thiogalactopyranoside (IPTG) at 18°C for 18 h. The cells were harvested by centrifugation at 6,500 rpm at 4°C for 20 min. The subsequent purification process for TgFer was performed as described by Ding et al. (2017). After the removal of the His_6_-SUMO tag using SUMO protease (at a 1:500 ratio of protease to protein), the protein was further purified using a HiLoad 16/600 Superdex 200 pg gel-filtration chromatography column (GE Healthcare, PA, United States). Then, the purified protein was subjected to 12% sodium dodecyl sulfate-polyacrylamide gel electrophoresis (SDS-PAGE), and the protein concentration was measured using a bicinchoninic acid (BCA) protein assay kit (Beyotime, Shanghai, China).

### Site-Directed Mutagenesis

The site-directed mutagenesis of TgFer was performed according to the instructions provided with the QuikChange Multi-site-directed mutagenesis kit (Agilent, CA, United States). The primers used for site-directed mutagenesis were shown in Supplementary Table S1. The TgFer variants D129A/E132A and E168A were verified by DNA sequencing. For the overproduction of TgFer variants, constructs were encoded in the pET-28a vector and transformed into *E. coli* BL21 (DE3) cells. The variant proteins were expressed and purified as described for the preparation of TgFer.

### Heavy Metal Copper Treatment

The purified proteins were diluted using buffer solution (25 mmol L^−1^ Tris–HCl, 150 mmol L^−1^ NaCl, pH 5.5). Twenty-milliliter portions of protein sample (approx. 0.5 mg mL^−1^) were dialyzed against 2 L of solution containing 2.5 mM CuCl_2_ at 4°C over 12 h as described by Ding et al. (2017). The protein sample was subsequently dialyzed in buffer solution, and the solution was replaced once every 4 h to remove free Cu^2+^ ions.

### Circular Dichroism Analysis

The protein samples (approx. 0.1 mg/ml) were prepared and transferred to a quartz cell with a 0.1 cm path length and 1.0 nm bandwidth. The Circular Dichroism (CD) spectra were recorded using a J-815 CD spectrometer (Japan Spectroscopic, Tokyo, Japan). The secondary structure of ferritins was analyzed by Yang’s method by Jasco-Corp (Tokyo, Japan), and the molar ellipticity [θ] (deg∙cm^2^∙dmol^−1^) was calculated as previously described (Ding et al., 2017).

### Inductively Coupled Plasma Mass Spectrometry Analysis

The protein samples (approx. 1.0 mg mL^−1^) were prepared and digested using microwave digestion (MARS 5, CEM Corp., Matthews, NC, United States), as described previously (Ding et al., 2017; Si et al., 2017). Metal ion content was then quantified with three replicates using a Thermo X Series II Inductively Coupled Plasma Mass Spectrometry (ICP-MS) instrument (Thermo Fisher Scientific Inc., MA, United States). The standard curves for Fe or Cu atom were obtained by using the multi-element standard solutions. The atoms per ferritin cage was calculated as described previously (Si et al., 2017).

### Crystallization, Data Collection, and Structure Determination

Preliminary screening for crystallization conditions was conducted using Crystal Screen I and II (Hampton Research, Riverside, CA, United States) through the sitting-drop vapor diffusion method. A volume of 1 µL protein solution (approx. 10 mgmL^−1^) in 25 mM Tris-HCl, 150 mM NaCl, pH 8.0 was combined with 1 µL reservoir solution in the well of a 48-well plate and incubated at 18°C. TgFer crystals suitable for X-ray data collection were obtained in 4.0 M sodium formate. TgFer + Cu crystals were obtained in a condition containing 0.2 M ammonium acetate, 0.1 M Tris hydrochloride, pH 7.5 and 30% (v/v) 2-propanol. The sample of TgFer loaded with ferrous ions (TgFer + Fe) was prepared by the addition of [(NH4)_2_Fe(SO_4_)_2_]·6H_2_O (Mohr’s salt) at a Fe^2+^ ion/TgFer molar ratio of 4,000:1 (150 mM HEPES, 150 mM NaCl, pH 6.5). TgFer + Fe crystals grew in solutions containing 0.1 M sodium cacodylate trihydrate, pH 6.5, 30% (v/v) (+/−)-2-Methyl-2,4-pentanediol. TgFer + CuFe crystals were obtained as reported previously by Pozzi et al. (2019). TgFer + Cu crystals experienced severe cracking after long exposure times to Mohr’s salt, limiting the iron diffusion to a maximum of 5 min. Thus, for TgFer + CuFe crystals, Mohr’s salt crystals were inserted directly into the crystallization drop containing TgFer + Cu crystals for a time-controlled exposure of 5 min. These crystals were harvested using a CryoLoop (Hampton Research). After soaking in a cryoprotection solution supplemented with 20% (v/v) glycerol, the crystals were immediately flash-cooled and stored in liquid nitrogen.

X-ray data were collected at the Shanghai Synchrotron Radiation Facility (SSRF) using a beamline BL17U1 (Shanghai, China) at 100 K, combined with a Quantum 315r CCD detector (Area Detector Systems Corp., CA, United States) (Wang et al., 2018a). The anomalous dispersion diffraction data for TgFer + Cu and TgFer + CuFe crystals were collected at the peak wavelength of X-ray absorption for Fe-K edge (*λ* = 1.7389 Å) (Pozzi et al., 2019). The diffraction data of TgFer and TgFer + Fe crystals were collected at a wavelength of 0.9789 Å. Diffracting data were indexed, integrated and scaled using the HKL2000 program suite (Minor et al., 2006). The resulting data were further processed using the CCP4 suite (Collaborative Computational Project, Number 4, 1994). The TgFer, TgFer + Cu, TgFer + Fe, and TgFer + CuFe structures were determined by molecular replacement with the coordinates of the published ferritins (PDB ID: 1BG7, 3A9Q, 1RCD, and 3A9Q, respectively) as the search model, using the program BALBES (Long et al., 2008). Manual model building was performed using the program COOT (Emsley and Cowtan, 2004), and automated model building and refinement were conducted using REFMAC5 (Murshudov et al., 2011). The positions of the iron or copper ions were determined based on anomalous difference Fourier maps and geometric parameters calculated using the program FFT (fast Fourier transform) from the CCP4 suite (Collaborative Computational Project, Number 4, 1994). Water molecules were positioned in self-defined residual densities by ARP/wARP (Cohen et al., 2004). The final models were inspected manually and checked with the programs COOT (Emsley and Cowtan, 2004) and PROCHECK (Laskowski et al., 1993). Data collection and final refinement statistics are given in Table 1.

**TABLE. 1:** Data collection and refinement statistics.

Crystal parameters	TgFer	TgFer + Cu	TgFer + Fe	TgFer + CuFe
Data collection				
Space group	C222	H32	P222	H32
a, b, c (Å)	214.6, 214.9, 151.9	217.0, 217.0, 132.9	181.9, 182.0, 182.0	217.3, 217.3, 134.1
α, β, γ (˚)	90, 90, 90	90, 90, 120	90, 90, 90	90, 90, 120
Wavelength (Å)	0.97892	1.73890	0.97892	1.73890
Resolution range (Å)	50.00–1.78 (1.81–1.78)*	50.00–2.30 (2.34–2.30)*	50.00–1.85 (1.88–1.85)*	50.00–3.90 (3.97–3.90)*
No. of reflections	330,028	51,876	505,323	10,532
Completeness (%)	98.9 (80.5)*	97.8 (100.0)*	99.7 (98.9)*	94.0 (73.4)*
I/σ(I)	13.8 (4.1)*	32.6 (21.4)*	11.3 (4.6)*	29.0 (4.0)*
R_merge_	0.366 (0.436)*	0.085 (0.218)*	0.364 (0.842)*	0.167 (0.523)*
Redundancy	6.7 (6.1)*	8.8 (8.6)*	8.7 (4.9)*	3.5 (3.1)*
CC_1/2_	0.987 (0.604)*	0.991 (0.981)*	0.986 (0.605)*	0.965 (0.884)*
Wilson B-factor (Å^2^)	8.99	27.86	11.59	81.08
Refinement				
R_work_/R_free_	0.204/0.231	0.195/0.208	0.196/0.262	0.264/0.306
Mean B-values (Å^2^)				
Protein	8.0	29.6	11.7	77.1
Ligand				
Water	2,311	401	2,719	1
Metal ion	27	45	108	8
R.m.s. deviations				
Bond lengths (Å)	0.005	0.006	0.006	0.003
Bond angles (˚)	0.781	0.734	0.732	0.503
Ramachandran plot (%)				
Favored region	98.7	98.2	98.7	98.5
Allowed region	1.2	1.8	1.3	1.4
Outlier region	0	0	0	0
PDB code	6L55	6KZY	6L56	6L58

*Highest resolution shell is shown in parenthesis.

### Protein Structure Analysis

Based on the amino acid sequence of TgFer, the theoretical isoelectric point (pI) and predicted molecular weight (MW) were deduced by the ExPASy server (http://web.expasy.org/peptide_mass/). The amino acid sequence searches for close homologs of TgFer were obtained from the GenBank database. A phylogenetic tree was constructed by the neighbor-joining method with 1,000 bootstrap replicates using MEGA 5.0 software (Tamura et al., 2011). Structure-based sequence alignment was performed using ClustalW (Sievers et al., 2011) and ESPript 3 server (Robert and Gouet, 2014). The superimposed structure of ferritins was performed using the Superpose online server (http://superpose.wishartlab.com/) (Maiti et al., 2004). All structure figures were generated using PyMOL molecular graphics system (Schrödinger LLC, https://pymol.org) (Schrödinger, 2018). The electrostatic potential on the molecular surface was calculated using the APBS plugin in PyMOL (Baker et al., 2001).

### Biochemical Assays

The purified TgFer (approx. 1.0 mgmL^−1^) was dialyzed against sample buffer (25 mM Tris-HCl, 150 mM NaCl, pH 5.5) containing 400, 800, 1,200, 1,600, 2000, or 2,400 µM CuCl_2_ (corresponding to a Cu^2+^ ion/TgFer molar ratio of 200:1, 400:1, 600:1, 800:1, 1,000:1, and 1,200:1, respectively) at room temperature for 30 min. Ferritin was then purified by buffer exchange using a PD-10 desalting column (GE Healthcare) to remove unbound Cu^2+^ ions. The interactions of TgFer with the different concentrations of Cu^2+^ ions were determined by ultraviolet–visible (UV–vis) absorption spectroscopy, ranging from 190 to 500 nm. UV–vis absorption spectra were measured using a Nanodrop 2000 spectrophotometer (Thermo Fisher Scientific Inc., MA, United States). All analyses were performed in three replicates with three recordings each.

The iron oxidation kinetics of TgFer, D129A/E132A and E168A mutants were examined by FlexA-200 full-wavelength Microplate reader (Hangzhou Allsheng Instruments Co., Ltd., Hangzhou, China) at 25°C, as previously described (Bou-Abdallah et al., 2019). Oxygen-free aliquots of Fe^2+^ samples (50 mM) were prepared by dissolving Mohr’s salt in 0.1% (v/v) HCl under anaerobic conditions. The protein samples were diluted anaerobically to a final concentration of approx. 1.0 mgmL^−1^. Reactions examining iron oxidation kinetics by protein samples were performed in 0.2 mM HEPES, pH 6.5, containing 150 mM NaCl, with molar ratios of Fe^2+^ ions to ferritin of 1,000:1 at 25°C (Toussaint et al., 2007). All kinetic experiments were repeated three times by independent protein preparations to ensure reproducibility. Iron (II) oxidation by each type of ferritin was monitored, despite a broad absorbance band, by measuring the initial 100 s of absorbance at 310 nm (McKnight et al., 1997; Toussaint et al., 2007).

To determine the iron storage ability of TgFer and TgFer + Cu, a freshly prepared solution of Mohr’s salt containing an Fe^2+^ ion/ferritin molar ratio of 4,000:1 was added to TgFer and TgFer + Cu solutions (150 mM HEPES, 150 mM NaCl, pH 6.5) at 25°C for complete reactions. The samples were then dialyzed in 2 mM HEPES with stirring, and the solution was replaced every 4 h. The obtained TgFer + Fe and TgFer + CuFe samples were loaded onto HiLoad 16/600 Superdex 200 pg gel-filtration chromatography column (GE Healthcare). The iron content was determined by ICP-MS, as described above.

### Statistical Analysis

Statistical analyses were performed using SigmaPlot 12.0 (Systat Software Inc., San Jose, California, United States), and significant differences were determined using a two-tailed Tukey’s honestly significant difference test, following an F-test. All data were plotted using Origin Pro 9.0 software (OriginLab, Northampton, MA, United States). All data are presented as the mean ± standard error of the mean (SEM), and a *p-*value of less than 0.05 or 0.01 was considered significant.

## Results

### Phylogenetic Analysis and Protein Characterization

**FIGURE 1:**
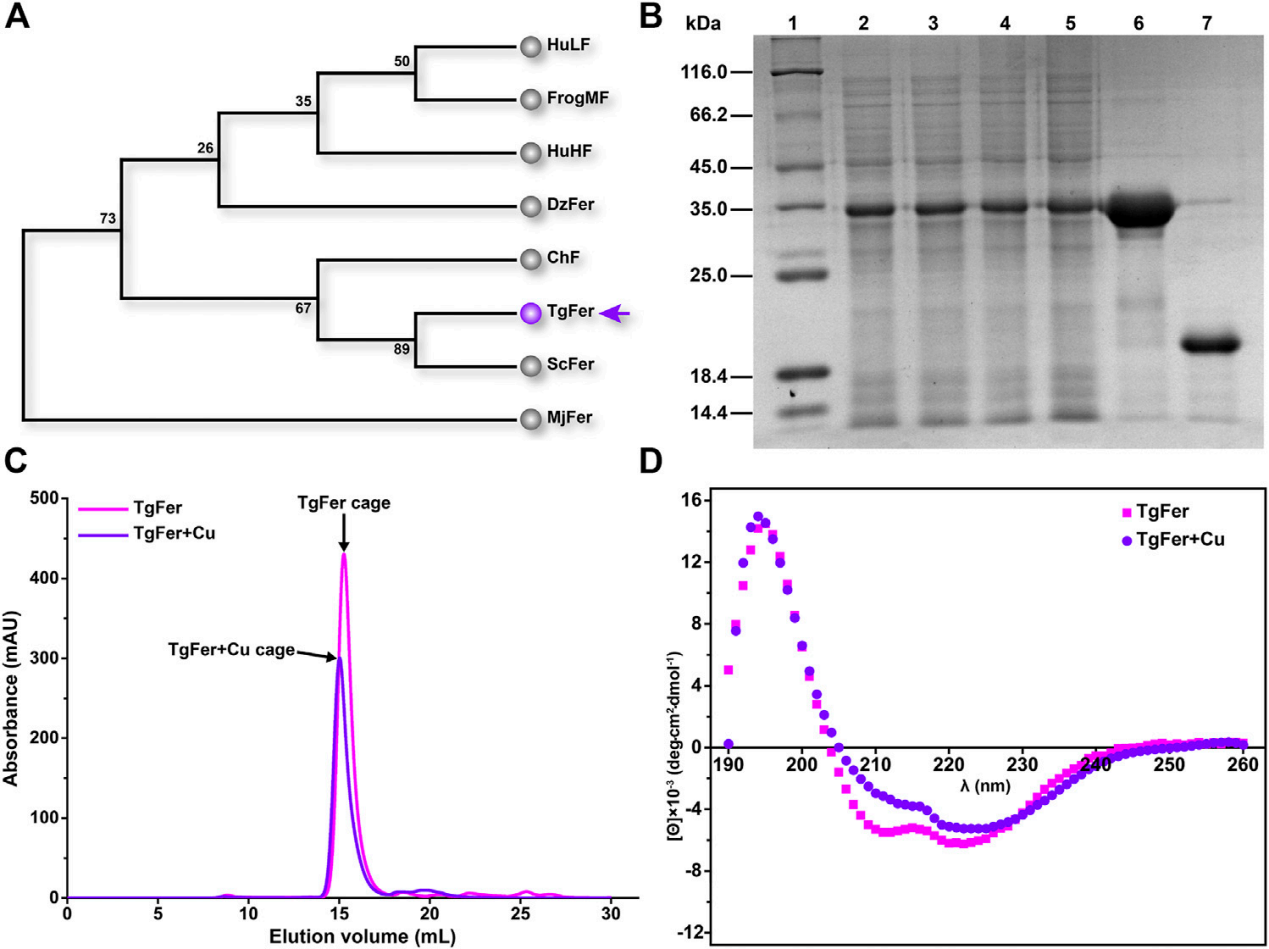
**(A)** Phylogenetic analysis was performed among eight ferritins from various species. Species abbreviations (with PDB accession ID in parentheses): TgFer, *Tegillarca granosa* ferritin (6L55); ScFer, *Sinonovacula constricta* ferritin (6LP5); ChF, *Chaetopterus* ferritin (5WPN); MjFer, *Marsupenaeus japonicus* ferritin (6A4U); DzFer, *Dendrorhynchus zhejiangensis* ferritin (7EMK); HuHF, human H-chain ferritin (2FHA); BfMF, bullfrog M ferritin (4DAS); HuLF, human L-chain ferritin (2FFX). **(B)** Expression and purification of recombinant TgFer. Lane 1: middle molecular marker; Lanes 2–3: total protein of TgFer induced 18°C for 14 h; Lanes 4–5: total protein of TgFer induced at 18°C for 18 h; Lane 6: purified TgFer protein; Lane 7: purified TgFer protein without SUMO tag. **(C)** Superdex 200 gel-filtration chromatography profile of TgFer and TgFer + Cu. The peak of the target protein is shown by a black arrow. **(D)** Circular dichroism spectra of TgFer and TgFer + Cu.

To investigate the evolutionary relationships between TgFer and ferritins from other representative organisms, a phylogenetic tree was constructed based on the deduced amino acid sequences of ferritins from eight species. As expected, TgFer falls within the cluster of Bivalvia (Mollusca) ferritins and is genetically closest to *Sinonovacula constricta* ferritin (ScFer) (Su et al., 2020) (Figure 1A). The predicted TgFer consists of 172 amino acids, with a predicted MW of 19.995 kDa and a theoretical pI of 4.89. SDS-PAGE analysis also suggested that the MW of the TgFer monomer was approximately 20 kDa, as shown in Figure 1B. After purification using a nickel-nitrilotriacetic acid (Ni-NTA) affinity column, the protein was further purified by gel filtration prior to crystallization, the sample was analyzed using a gel-filtration chromatography column, resulting in a peak at approx. 15 ml, which is consistent with the TgFer 24-mer (Figure 1C). CD measurements showed that both TgFer and TgFer + Cu were highly *α*-helical, with similar spectral features of well-defined minima at 211 and 222 nm and a maximum at 194 nm (Figure 1D).

### Structure Determination

**FIGURE 2:**
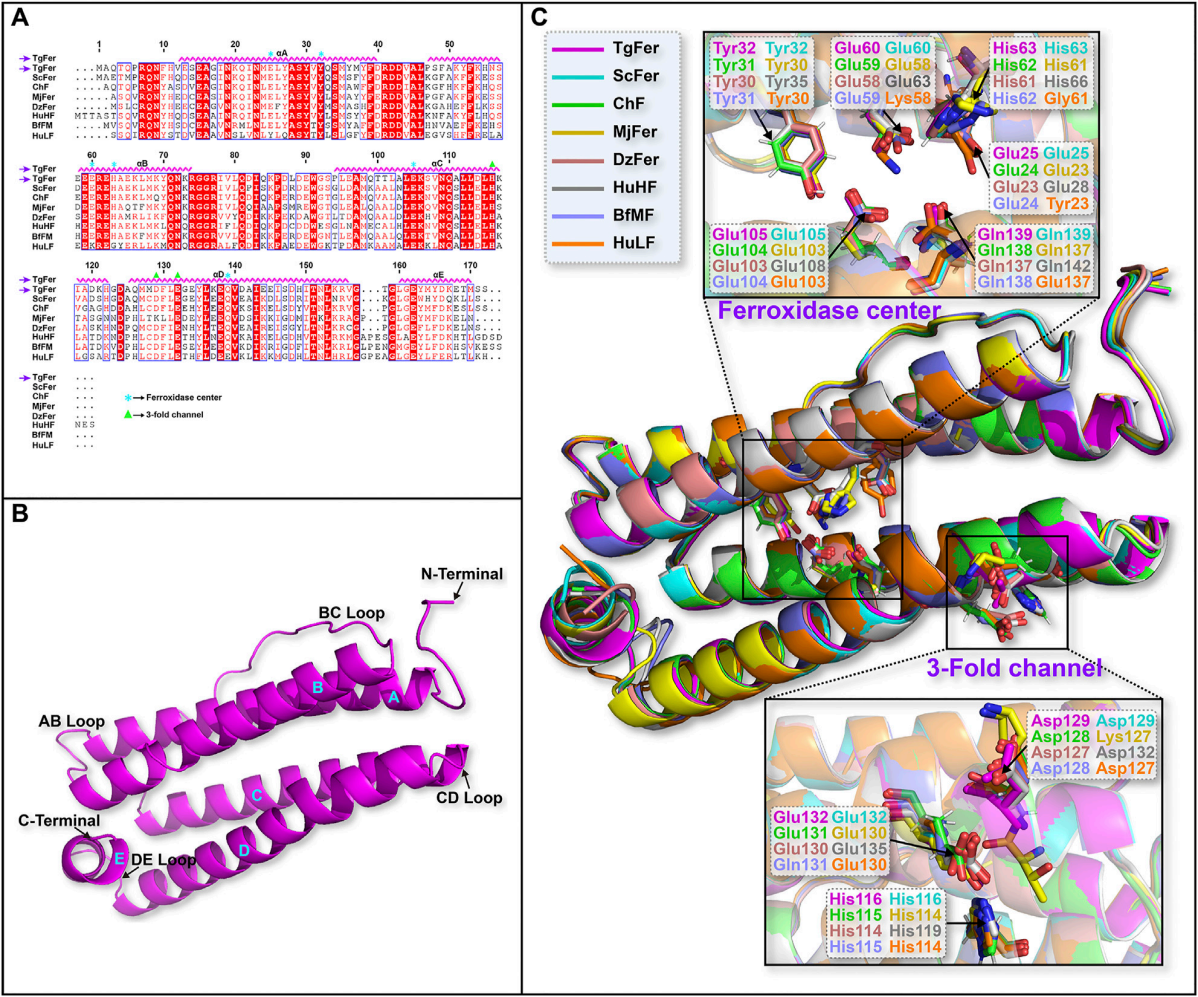
**(A)** Multiple sequence alignment of TgFer compared with ferritins from other species. **(B)** Cartoon representation of the TgFer subunit. **(C)** Structure superposition of TgFer and the other seven ferritins.

Next, we searched for TgFer homologs using BLAST. Structure-based sequence alignment indicated that TgFer and other ferritins were highly conserved among the examined species, sequence identities ranging from 45.7 to 76.6% (Figure 2A). The closest sequence to that of TgFer was found for ScFer, expressed by the marine invertebrate *S. constricta,* with a sequence identity of 76.6% (Su et al., 2020). Although TgFer and human L-chain ferritin (HuLF) share a relatively low sequence identity of 45.7% (Wang et al., 2021), they share highly conserved ferroxidase sites and 3-fold channels, similar to other ferritins (Figures 2A,C).

In this study, the crystal structure of TgFer was solved by molecular replacement and contained a dodecamer in the asymmetric unit. The final model was refined at a resolution of 1.78 Å and featured a visible electron density for residues 3–171 of each subunit, resulting in an R_work_ of 0.20 and an R_free_ of 0.23 (Table 1 and Figure 2B). As shown in Figure 2B, each of TgFer subunits is characterized by a four-α-helix bundle containing two antiparallel helix pairs (A, B and C, D) and a fifth short E-helix that forms an angle of approx. 60° with respect to the four-helical bundle. Four helices comprise residues 12–40 (helix A), 47–74 (helix B), 94–122 (helix C), and 125–156 (helix D). A short, C-terminal α-helix (helix E, residues 160–170) stems from one end of helix D at the C-terminal end, similar to that observed for other ferritin family proteins (Figures 2A,C).

### Structure Determination

**FIGURE 3:**
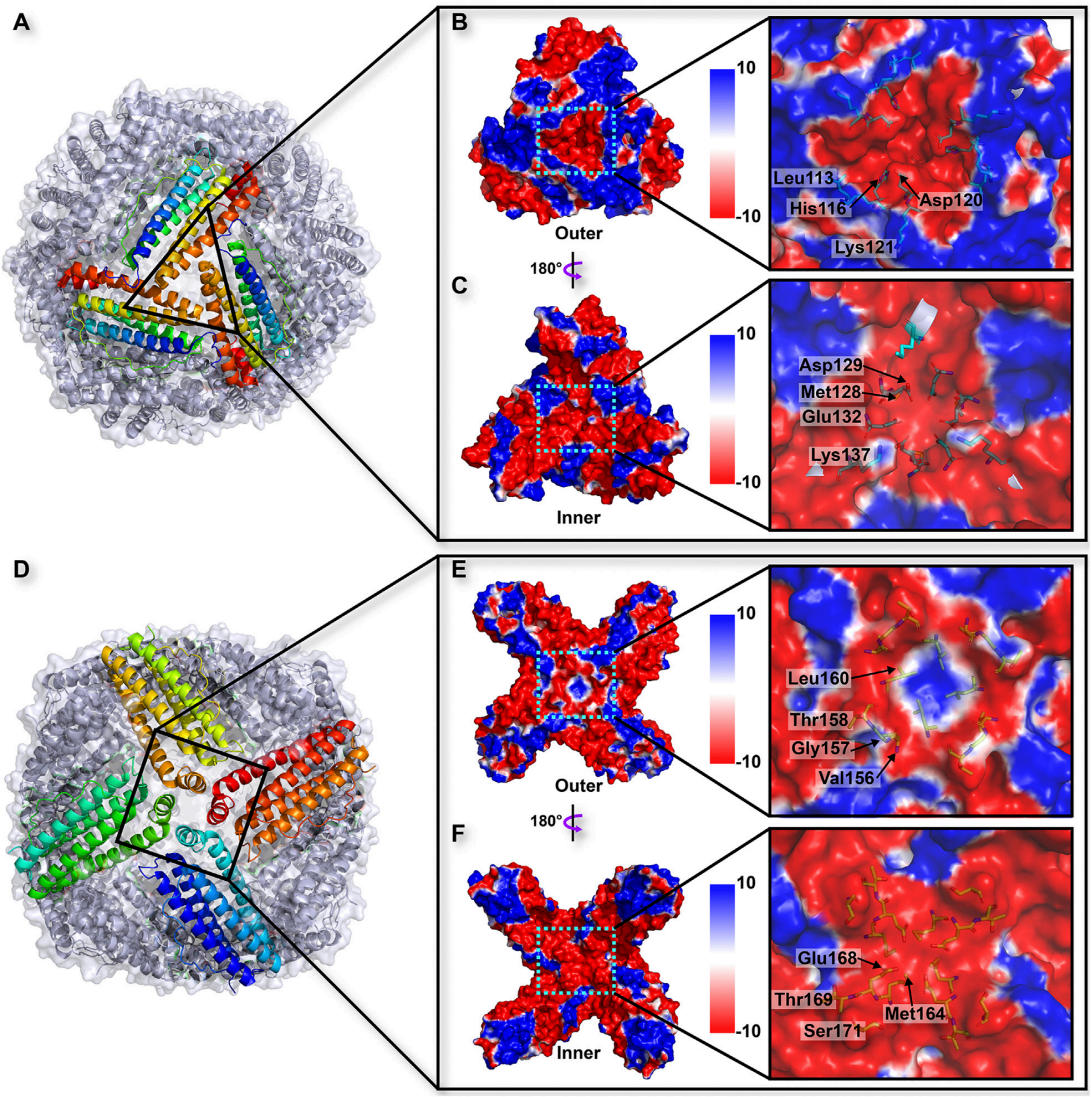
**(A)** Overall structure related to the trajectory of iron entry into the TgFer nanocage through the 3-fold channel. The electrostatic surface potential from outside **(B)** and inside **(C)** of the nanocage of the 3-fold channel. **(D)** Overall structure of TgFer nanocage through the 4-fold channel. The electrostatic surface potential from outside **(E)** and inside **(F)** of the cage at the 4-fold channel. The potential scale is rendered from the −10 kTe^−1^ to +10 kTe^−1^ from red to blue.

Similar to the structures of most ferritins obtained from vertebrates, the crystal structure of TgFer in marine invertebrate exhibits a cage-like spherical shell composed of 24 identical subunits, resulting in arrangements of 4-, 3-, and 2-fold symmetry (Figure 3). TgFer is a porous protein containing eight 3-fold channels and six 4-fold channels that traverse the shell of the 24-mer cage-like structure (Figures 3A,D). Electrostatic potential calculations showed that the outer entrance to the 3-fold channel is conspicuously surrounded by negative potential values across the whole region, which extend across the protein shell to the inner entry (Figures 3B,C). Major contributors to the conspicuously negative potential over nearly the entire 3-fold channel include the highly conserved residues Asp129 and Glu132. Our calculations showed that the 4-fold channel was surrounded by the negative potential of the annular region, whereas the potential on the outer entrance of this channel tends to be positive or neutral, as demonstrated in Figure 3E. The prevalence of negatively charged regions is significantly dominant in the inner entrance of the 4-fold channel (Figure 3F). The potential on the exterior and interior surface of the 4-fold channel is generally negative, with only a few regions featuring positive values, mainly attributed to a negatively charged residue Glu168 and the carbonyl groups at the C-termini of four α-helices involving neutrally charged residues (Val156, Thr158, Met164, Thr169, and Ser171) (Figures 3E,F).

**FIGURE 4:**
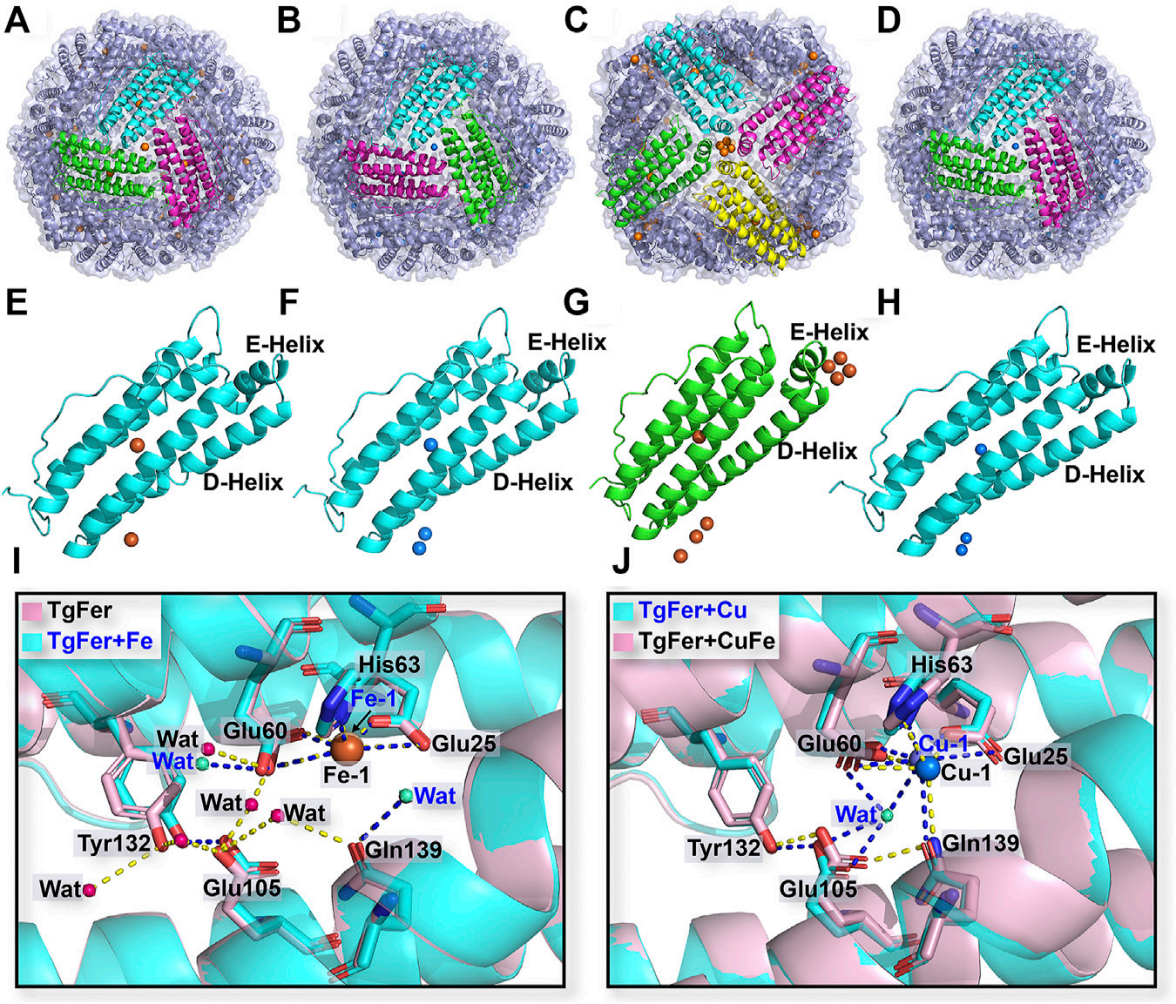
Overall structure of TgFer **(A)**, TgFer + Cu **(B)**, TgFer + Fe **(C)**, and TgFer + CuFe **(D)**. A single ferritin subunit of TgFer **(E)**, TgFer + Cu **(F)**, TgFer + Fe **(G)**, and TgFer + CuFe **(H)**, in which yellow and blue spheres represented iron and copper ions, respectively. **(I)** The detailed structure of the ferroxidase site view, showing the coordination environment of iron ions (yellow spheres) in TgFer and TgFer + Fe. **(J)** The detailed structure of the coordination environment of copper ions (blue spheres) at the ferroxidase site in TgFer + Cu and TgFer + CuFe.

The crystal structures of TgFer + Cu, TgFer + Fe, and TgFer + CuFe, containing iron or copper ions, were determined with resolutions of 2.30, 1.85, and 3.90 Å, respectively (Table 1). After the restrained refinement of the final model, they refined to crystallographic R_work_/R_free_ of 0.152/0.195, 0.165/0.196, and 0.187/0.264, respectively. Consistent with the structure of TgFer, the overall structures of TgFer + Cu, TgFer + Fe, and TgFer + CuFe were identified as 24-mer hollow cages (Figures 4A–D). The structural superimposition of the TgFer subunit with the TgFer + Cu, TgFer + Fe, and TgFer + CuFe subunits led to a root-mean-square deviation (RMSD) value of 0.36 Å over 168 Cα atoms, 0.20 Å over 169 Cα atoms, and 0.16 Å over 168 Cα atoms, respectively (Figures 4E–H).

### Structures of the Ferroxidase Site and 3-Fold Channel

Based on the multiple sequence alignments, the ferroxidase site of TgFer is composed of highly conserved amino acids, particularly the six residues Glu25, Tyr32, Glu60, His63, Glu105, and Gln139 (Figure 2A). The resulting TgFer structures reveal that only one Fe^2+^ ion (Fe-1) is bound to the ferroxidase site with occupancy of 80%, where it is coordinated with the OE1 of Glu25 (helix A), the OE1 of Glu60 (helix B), the ND1 of His63 (helix B) and two waters (Figure 4I and Supplementary Figure S1A). The Fe-binding position in the ferroxidase site of TgFer + Fe is consistent with that of TgFer (Figure 4I and Supplementary Figure S1C). In TgFer + Cu, the ferroxidase binding site appeared to be occupied by one Cu^2+^ ion (90% occupancy) (Figure 4J) because no peaks corresponding to an Fe^2+^ ion were identified at this site, based on an anomalous difference Fourier map (Supplementary Figure S1B, Supplementary Figure S2A). After incubation with Fe^2+^ ions, the site containing the Cu^2+^ ion was not replaced with an Fe^2+^ ion (Figure 4J), and this was confirmed based on the anomalous difference Fourier map (Supplementary Figure S1D, Supplementary Figure S2B).

**FIGURE 5:**
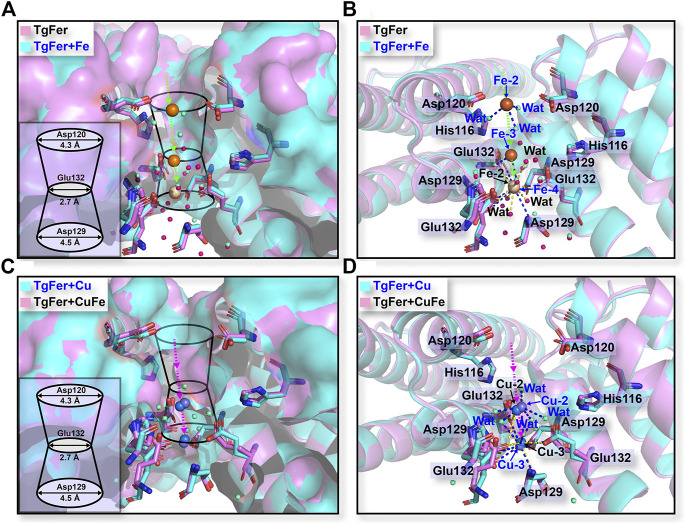
**(A)** Stereo views of the 3-fold channels for TgFer and TgFer + Fe. **(B)** The detailed structure of the coordination environment for iron ions at the 3-fold channel in TgFer and TgFer + Fe (side view). Yellow spheres represent iron ions. **(C)** Stereo views of the 3-fold channels for TgFer + Cu and TgFer + CuFe. **(D)** The detailed structure of the coordination environment for copper ions at the 3-fold channel in TgFer + Cu and TgFer + CuFe (side view). Blue spheres represent copper ions.

**FIGURE 6:**
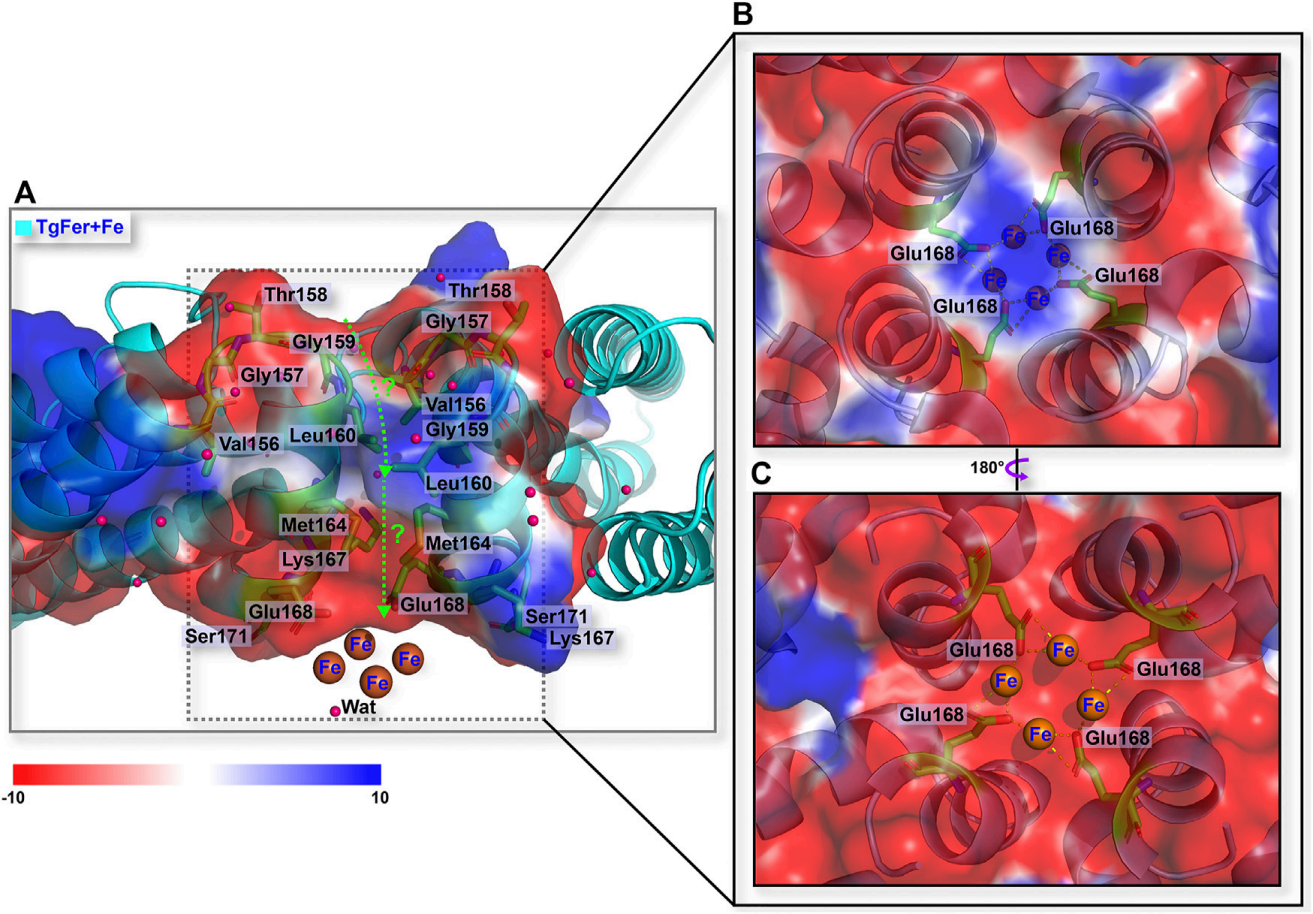
**(A)** Schematic overview of the 4-fold channel of TgFer + Fe. **(B)** The detailed coordination environment for iron ions viewed from the outside of the 4-fold channel in TgFer + Fe. **(C)** The detailed coordination environment for iron ions from the inside view of the 4-fold channel in TgFer + Fe. The pink sphere represents water molecule.

In TgFer, the hydrophilic channel penetrating along the 3-fold symmetry channel is considered to represent one potential Fe^2+^ ion entry channel to the ferritin cavity. One Fe^2+^ ion (Fe-2) with an occupancy of 60% is coordinated by the three OEs of Glu132 residues (distance of 2.63 ± 0.08 Å) from all three symmetrical subunits and three water molecules (Fe-2–Wat1 2.91 ± 0.11 Å, Fe-2–Wat2 2.73 ± 0.13 Å, and Fe-2–Wat3 2.83 ± 0.12 Å distances) (Figure 5B and Supplementary Figure S3A). In the TgFer + Fe structure, three Fe^2+^ ions form into a metal wire in the 3-fold channel (Figure 5A). The Fe-2 and Fe-3 ions (intermetallic distance Fe-2–Fe-3 of 6.10 ± 0.09 Å) with occupancies of 60 and 70% respectively are each coordinated by three water molecules (Figure 5B and Supplementary Figure S3C). The Fe-4 ion (80% occupancy) is coordinated by the three OEs of Glu132 residues (distance of 2.75 ± 0.24 Å) from all of the 3-fold symmetry subunits and three water molecules (Fe-4–Wat1 3.12 ± 0.14 Å, Fe-4–Wat2 3.07 ± 0.08 Å, and Fe-4–Wat3 3.17 ± 0.09 Å distances), while the averaged Fe-3–Fe-4 distance is 4.30 ± 0.10 Å. In addition, the 4-fold pores of the TgFer cage are defined by the 4 E helices from symmetry-related subunits, forming a four-helix bundle to which four Fe^2+^ ions (90% occupancy) are bound between the negative potential pore layer formed by Glu168 residues (Figure 6 and Supplementary Figure S4A). Four iron ions ligand environment inside the 4-fold channel is completed by the eight OEs of Glu132 residues at distances of 3.10 ± 0.13 and 3.15 ± 0.11 Å and a water molecule with the Fe–Wat distance of 3.97 ± 0.18 Å (Supplementary Figure S4B). In the TgFer + Cu structure, two Cu^2+^ ions are found in the 3-fold channel (Figure 5C). The Cu^2+^ ion (Cu-2) is located practically outside the channel bridged with three water molecules, and the intermetallic distance Cu-2–Cu-3 in the sites is 3.74 ± 0.23 Å (Figure 5D and Supplementary Figure S2C). The other Cu^2+^ ion (Cu-3) is located deep inside the channel, coordinated with the OD1 from Asp129 residue (distance of 2.71 ± 0.22 Å) and the OE2 from Glu132 residue (distance of 2.64 ± 0.36 Å) from the 3-fold symmetrical subunits (Supplementary Figure S2C). The occupancies for Cu-2 and Cu-3 in these sites have been estimated to 80 and 90%. The metal ions at these sites were confirmed as being Cu^2+^ ions by an anomalous difference electron density map (Supplementary Figure S3B). Similar to the crystal structure of TgFer + Cu, two Cu^2+^ ions with occupancies of 90% in TgFer + CuFe are bound to the 3-fold channel based on the anomalous difference electron density map (Figure 5D and Supplementary Figure S3D). One copper ion (Cu-2) on the outer surface of the ferritin shell had six coordinated waters with the Cu–Wat distance of 2.52 ± 0.20 Å, and the intermetallic distance Cu-2–Cu-3 in the sites is 4.25 ± 0.34 Å (Figure 5D and Supplementary Figure S2D). The other copper ion (Cu-3) positioned deeper inside the 3-fold symmetry channel is coordinated by the OD1 from Asp129 residue (distance of 2.68 ± 0.02 Å) and the OE2 from Glu132 residue (distance of 2.78 ± 0.04 Å) (Supplementary Figure S2D).

### Biochemical Property of the Protein Samples

**FIGURE 7:**
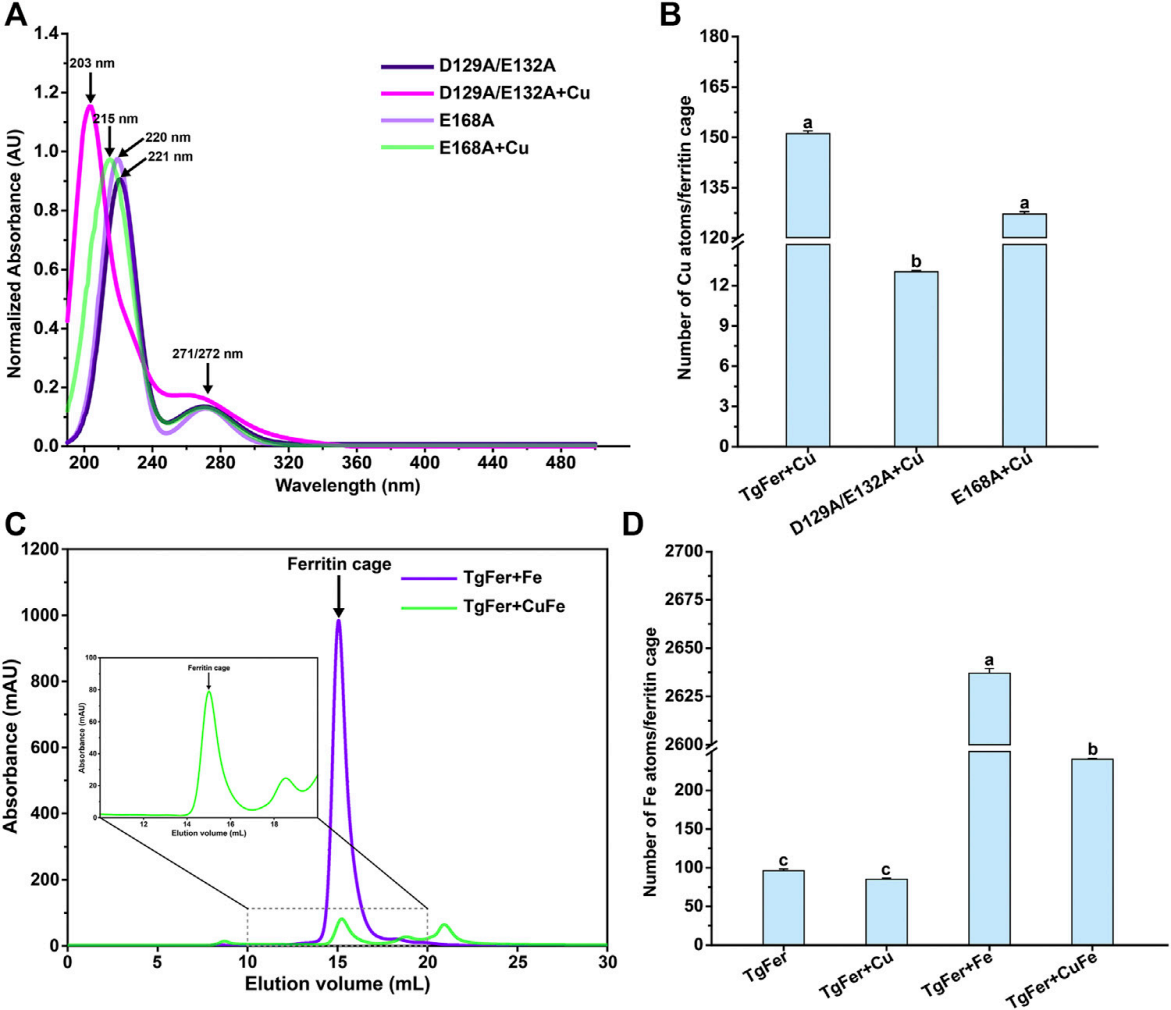
**(A)** UV–vis absorbance spectrum for E168A and D129A/E132A and those proteins of treated with Cu^2+^ ions. **(B)** The determination of copper atom contents in different variant ferritin treatment groups by ICP-MS. **(C)** Gel-filtration chromatography profile for TgFer + Fe and TgFer + CuFe. The peak of the target protein is marked with a black arrow. **(D)** The determination of iron atom contents in different ferritin treatment groups by ICP-MS. The values are indicated as the mean ± SEM (*n* = 3).

Of note, the two acidic residues Asp127 and Glu130, corresponding to Asp129 and Glu132 residues in TgFer, had been proven to exert electrostatic attractions on the incoming metal ions in *Rana catesbeiana* bullfrog M ferritin (RcMf) variants (Bernacchioni et al., 2016) and the wild-type RcMf protein (Chandramouli et al., 2016). Moreover, the residue Glu167, corresponding to Glu168 residue in TgFer, had been considered as a critical metal coordination site at the 4-fold channel in *Chaetopterus* ferritin (ChF) (De Meulenaere et al., 2017). To assess the functions of the D129/E132 and E168 residues of TgFer, Asp129 and Glu132 residues from the 3-fold channel and Glu168 residue from the 4-fold channel were each replaced with alanine residues by site-specific mutagenesis. The D129A/E132A and E168A mutants are prepared using an *Escherichia coli-*based expression system. To determine the roles played by these two variant proteins in Cu^2+^ ion uptake, they were treated with copper ions as described above. As shown in Figure 7A, the UV–vis spectrum of D129A/E132A + Cu showed a corresponding blue shift of 18 nm (from 221 to 203 nm) compared with the D129A/E132A peak, whereas the characteristic absorption peak at 280 nm almost disappeared. Similarly, the UV–vis spectrum of E168A + Cu exhibited a slight blue shift of 5 nm (from 220 to 215 nm) compared with that of E168A. ICP-MS analysis was used to further determine the copper contents of both D129A/E132A + Cu and E168A + Cu (Figure 7B). The copper content of TgFer + Cu was determined to be approximately 151 ± 0.83 atoms per cage. In contrast, the D129A/E132A mutant demonstrated a significant decrease, with a copper content of only 13 ± 0.06 (*p* < 0.05) atoms per cage, whereas the E168A mutant demonstrated only a slight decrease, with the copper content of 127 ± 0.72 (*p* > 0.05) atoms per cage. After the separate enrichment of iron for TgFer and TgFer + Cu, the target proteins were further purified by gel filtration, resulting in the elution of TgFer + Fe and TgFer + CuFe as symmetric peaks at approximately 15 ml on a calibrated gel-filtration chromatography column (Figure 7C). As shown in Figure 7D, the iron contents of the purified TgFer and TgFer + Cu proteins were determined to be approximately 96 ± 2.16 and 85 ± 1.25 atoms per cage, respectively, based on ICP-MS analysis. Similarly, significant differences in the iron contents were observed between TgFer + Fe and TgFer + CuFe proteins, which were measured at 2,637 ± 2.46 and 240 ± 0.97 atoms per cage, respectively.

**FIGURE 8:**
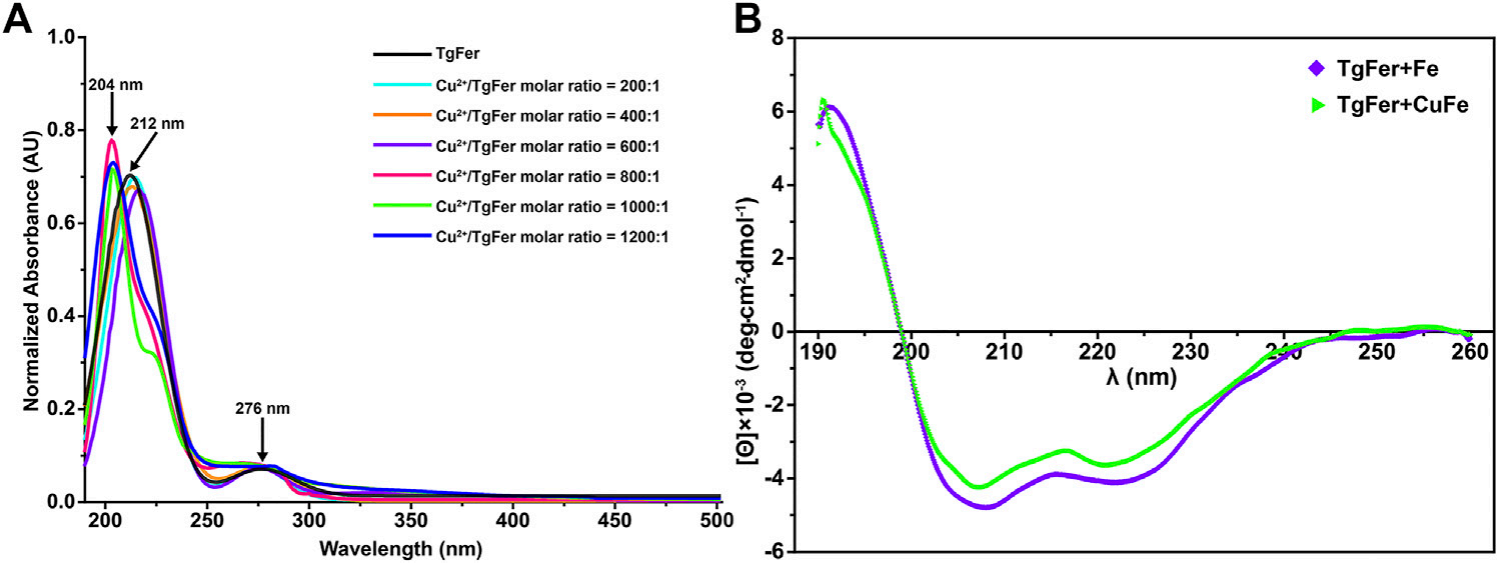
**(A)** UV–vis absorption spectra of TgFer samples treated with Cu^2+^ ions. Cu^2+^/TgFer molar ratios ranging from 200:1 to 1,200:1. The peaks corresponding to ferritins are indicated by a black arrow. **(B)** Circular dichroism spectra of TgFer + Fe and TgFer + CuFe.

As shown in Figure 8A, the absorbance spectrum of TgFer and TgFer + Cu proteins had two maximal absorption peaks; one was the protein characteristic absorption peak at about 280 nm and the other showed a distinct blue shift of 8 nm (from 212 to 204 nm) with the increase of Cu^2+^ ion/TgFer molar ratio. The CD spectra of TgFer, TgFer + Cu, TgFer + Fe, and TgFer + CuFe showed that all proteins had positive and negative absorption peaks in the 190–198 and 198–254 nm wavelength ranges (Figure 8B). To verify the iron oxidation ability of D129A/E132A and E168A mutants, the iron oxidation kinetics of them were determined by monitoring the concentration of oxidized iron inside ferritin at 310 nm. The progress curves of iron oxidation kinetics for the D129A/E132A and E168A mutants were presented in Supplementary Figure S4C. The result indicated that the D129A/E132A mutant had a greater effect on the iron oxidation rate, showing obviously weakened oxidation activity compared with TgFer, whereas the E168A mutant displayed not significantly different from that of TgFer.

## Discussion

Copper is commonly viewed as an essential trace element that is required for all living cells. Typically, copper can act as an active cofactor for many proteins that participate in biological processes, such as antibacterial activity and the disposal of toxic reactive oxygen species, due to its ability to cycle between redox states (Duncan and White, 2011; Rubino and Franz, 2012). However, excess copper can be highly toxic to cells due to its reactivity with molecular oxygen (Macomber and Imlay, 2009). Ferritin has been proposed not only to play a crucial role in the iron accumulation, but also to be as a nanocontainer for a number of metal ions such as Cu^2+^ ion, etc., (Maity et al., 2019). However, the molecular mechanisms underlying the ferritin-mediated effects on iron and copper trafficking remain poorly understood. Here, we heterologously expressed and purified TgFer and performed detailed structural and biochemical characterizations on TgFer expressed by the benthic marine invertebrate blood clam *T. granosa,* which is a member of the bivalve shellfish family.

The phylogenetic tree analysis revealed that TgFer is closely related to ScFer (Figure 1A). The amino acid sequence alignment suggested that TgFer possesses moderate amino acid sequence identity with orthologous proteins from six different organisms (approx. 60.5–76.6%), although the lowest sequence identity was shared with HuLF (approx. 45.9%) (Figure 2A). Structural comparisons indicated that TgFer monomers and those of other ferritins superimpose with an RMSD of 0.42–0.66 Å over 168 or 169 Cα atoms, and the superposition of TgFer with HuLF resulted in an RMSD of 0.66 Å over 169 Cα atoms (Figure 2C). Aside from HuLF, TgFer, compared with the other six ferritins from different organisms, revealed highly conserved and similar amino acid sequences and organization for the ferroxidase center and 3-fold channel. Our calculations indicate that the entire 3-fold channel is marked by the presence of negatively charged residues (Figures 3B,C), which appear to direct irons into the cavity, as previously reported for other marine invertebrate ferritins (Wang et al., 2021; Ming et al., 2021). Interestingly, only a small cluster of positive charges exists on the exterior of the 4-fold channel, mainly attributing to the NH groups at the N-termini of four α-helices from less conserved Met164 residue (Figure 2A and Figure 3F) similar to those observed in human H-chain ferritin (HuHF) (Douglas and Ripoll, 1998). The electrostatic potential of the internal region surrounding the 4-fold channel is dominated by the predominance of negative charges, attributed to the carbonyl groups at the C-termini of four α-helices from less conserved Met164, Glu168, Thr169, and Ser171 residues, similar to the electrostatic potential distribution of HuLF (Levi and Rovida, 2015). The TgFer structure B-factors suggested that these regions were very flexible. Although the distribution of the electrostatic potential of the 4-fold channel appears to provide an accessible pathway for cations (e.g., HuLF) (Wang et al., 2021), it is hard to determine whether the 4-fold channels in TgFer are involved in the entry of iron.

To reveal differences between similarly structured proteins, TgFer and TgFer + Cu were analyzed by spectroscopic techniques. Compared with TgFer, we observed a change in the secondary structure of TgFer + Cu, including an approx. 9.3% increase in α-helical content and the decreased contents in *β*-turn (approx. 3.8%) and random coil (approx. 5.5%) (Figure 1D and Supplementary Table S2). In comparison, for TgFer + Fe and TgFer + CuFe, the content of α-helix and *β*-turn in proteins decreased while β-sheet and random coil increased to different extents (Figure 8B and Supplementary Table S2). Previous research revealed that Cu^2+^ ions highly preferred to bind to the imidazole ring of His residue (Huard et al., 2013; Fragoso et al., 2013), possibly resulting in subtle changes in protein conformation. Therefore, it can be concluded that Cu^2+^ ions are likely to be bound tightly to the TgFer capsid, resulting in a constitutive change in the local conformation of TgFer + Cu. To confirm this hypothesis, we utilized a different method to profile the metal-binding sites and obtain detailed information. Here, we established precise, three-dimensional, structural models using comparative analyses of X-ray crystal structures to reveal structural differences. The results showed that the overall structures of TgFer, TgFer + Cu, TgFer + Fe, and TgFer + CuFe were highly conserved (Figure 4), similar to previously identified ferritin structures (Su et al., 2020; Ming et al., 2021). In the crystal structures of TgFer and TgFer + Fe, only site A of the ferroxidase center was occupied by Fe-1, but no clear electron density indicative of a metal ligand was visible around site B (Figure 4I), similar to the structural features of *Marsupenaeus japonicus* ferritin (MjFer) (Masuda et al., 2018). By contrast, we also observed that merely one copper ion (Cu-1) was present at site A of the ferroxidase site in TgFer + Cu and TgFer + CuFe (Figure 4J). Among them, the copper-binding amino acid residues were consistent with the bullfrog M ferritin (BfMF) crystal structure (Bertini et al., 2012), which shared the same ligands (Glu and His residues), with the exception of the Gln residue. Cu^2+^ ions have been demonstrated to display special coordination properties, such as the ability of Cu^2+^ ion to coordinate strongly with the imidazole nitrogens (Fragoso et al., 2013), and Cu^2+^ ion has been characterized as an inhibitor of the catalyzed reactions between Fe^2+^ ion and dioxygen in ferritin (Bertini et al., 2012). Thus, these results lead us to propose that copper binding to the ferroxidase site can potentially block iron binding to inhibit the ferroxidase activity in TgFer.

Numerous studies have implied that the 3-fold channel of ferritins can serve as a potential gateway for the entry of metal ions into the cage (Maity et al., 2019; Ming et al., 2021). Generally, the 3-fold channel can form a constriction in the middle like an hour-glass rather than being shaped like cylindrical tubes, as highlighted by Figures 5A,C. Consistent with the reported crystal structures of RcMf (Tosha et al., 2010), the 3-fold channel of TgFer is a variable width with the narrowest dimension of 2.7 Å caused by the carboxylate side chains of three conserved Glu132 residues from D-helices. Furthermore, two rings comprised of two negatively charged amino acids (Asp and Glu residues) in the 3-fold channel have also been demonstrated to strongly interact with divalent iron ions (Laghaei et al., 2013). Just as previously reported by the work of the groups of Pozzi and Mangani, they have demonstrated a doubly occupied native 3-fold channel by Fe^2+^ hexaaqua ion ([Fe(H_2_O)_6_]^2+^) that interacts with the side chains of Asp127 and Glu130 residues in human H ferritin (HuHF) (Pozzi et al., 2015a) and RcMf (Pozzi et al., 2015b). However, it is rather hard for fully hydrated Fe^2+^ ions with 6.4–6.9 Å in diameters to pass through the Glu130 constriction (approx. 2.3 Å diameter) in the 3-fold channel of RcMf (Behera and Theil, 2014). Therefore, either partial/full dehydration of [Fe(H_2_O)_6_]^2+^ must occur for ion passage through 3-fold channel, resulting in a direct coordination between the metal ions and Asp/Glu residues in order for the Fe^2+^ ions to reach the ferroxidase center in ferritin (Behera and Theil, 2014; Behera et al., 2015). Moreover, De Meulenaere et al. (2017) demonstrated that the conserved Glu131 residue at the 3-fold channel had been identified as a critical metal binding site in the Zn-bound structure of ChF. Wang et al. also revealed that Hg^2+^ ion could coordinate directly with the residues nearby its 3-fold channel of MjFer, largely impeding the entrance of ferrous ions (Wang et al., 2021). Besides, several reports have suggested that the metal ions can conventionally bind to the highly conserved Asp and Glu residues within the 3-fold channel from the marine invertebrates *S. constricta* ferritin (ScFer) (Su et al., 2020), *Phascolosoma esculenta* ferritin (PeFer and Fer147) (Ming et al., 2021) and *Dendrorhynchus zhejiangensis* ferritin (DzFer) (Huan et al., 2020).

For TgFer and TgFer + Fe, the free iron ions outside of ferritin can be attracted by the His116 and Asp120 residues located at the entrance of the 3-fold channel, then guided into the 3-fold pore, where they interact with the side chains of Asp129 and Glu132 residues, as illustrated in Figures 5A,B, indicating the crucial role played by the unique electrostatic arrangement of the 3-fold channel in iron transportation (Maity et al., 2019). It has been proposed that the Fe^2+^ ion located at the deepest part of the channel pore can travel toward the ferroxidase center with the help of amino acid residue (Chen et al., 2020). In TgFer + CuFe, two Cu^2+^ ions were tightly coordinated with the Asp129 and Glu132 residues of the 3-fold pore, similar to the structure of TgFer + Cu, and the Cu-Cu distance was approximately 4.5 Å and 3.9 Å, respectively (Figure 5D and Supplementary Figure S3). The observed change in the copper-binding ability of the D129A/E132A mutant strongly suggested that these two residues play crucial roles in metal ion adsorption (Figure 7B). The results revealed that copper could be able to compete with iron for uptake in TgFer + CuFe, likely due to the preferential binding of the Cu^2+^ ion with His residue (imidazole nitrogen) over the iron ion (Fragoso et al., 2013; Tan et al., 2014).

The iron transit efficiency *via* 3-fold channel can be monitored by determining the kinetics of the catalytic oxidation reaction occurring at the ferroxidase centers in ferritin (Chandramouli et al., 2016). To elucidate the effects of mutations on the 3- and 4-fold channel, the iron oxidation kinetics of D129A/E132A mutant were determined indirectly by monitoring the concentration of oxidized iron inside ferritin at 310 nm (Toussaint et al., 2007) (Supplementary Figure S4C). We found that the D129A/E132A mutant around 3-fold channel had a marked effect on the Fe^2+^ ion oxidation kinetics, while E168A mutant inside 4-fold channel has little effect on the oxidation rates. Similar results were observed with the mutations around the 3-fold channel of HuHF (Levi et al., 1996) and RcMf (Haldar et al., 2011). It is commonly thought that the Asp131 and Glu134 residues located at the narrowest part of the 3-fold channel in HuHF, corresponding to the Asp129 and Glu132 residues for TgFer, are of great importance during the iron uptake process (Pozzi et al., 2015a). Previous research also revealed that when the hydrophilic Asp and Glu residues were substituted by hydrophobic Ala residue, ferrous ions were inhibited from binding the 3-fold channel, resulting in the low speed of Fe^2+^ transport into the cavity, and thus to greatly affect the ferroxidase activity (Levi et al., 1996; Jin et al., 2019). Moreover, Haldar et al. reported that all of D127A and E130A mutants could influenced catalysis significantly, suggesting that Fe^2+^ ions were captured by Glu130 residue first and then directed to active sites by Asp127 residue in frog ferritin (Haldar et al., 2011). Collectively, as the narrowest part of the 3-fold channel in TgFer, Asp129 and Glu132 residues are of great importance during the iron uptake process. Thus, it can be concluded that Cu^2+^ ion binding at 3-fold channel may affect the function of iron uptake in the structure of TgFer.

The structural analyses indicate that the 4-fold channel of TgFer presents the special electrostatic arrangement (Figures 3E,F). Although four iron-binding sites are coordinated by four Glu168 residues in the TgFer + Fe structure (Figure 6 and Supplementary Figure S4), similar to that previously reported His173 residue for RcMf (Pozzi et al., 2015a), the E168A mutant has little effect on the oxidation rates compared with TgFer (Supplementary Figure S4C). Nevertheless, unlike His173 residue in HuHF (Laghaei et al., 2013) and RcMf (Pozzi et al., 2015a), the metal-binding residue Glu168 of TgFer is highly conserved with that of Glu residue from some marine invertebrate ferritins (Figure 2A). The Glu168 residue in TgFer is equivalent to the Glu166 residue in MjFer (Masuda et al., 2018) and the Glu167 residue in ChF (De Meulenaere et al., 2017) and can be used as a crucial binding site of metal ion at the 4-fold channel, as verified by the E168A mutant (Figure 7B) and shown in Supplementary Figures S4A, S4B. However, there is still in lack of sufficient evidence that whether the 4-fold channel is strongly correlated with metal transit, e.g., associated with iron uptake, ferroxidase activity, and many others.

In this study, we also find that there are obviously distinct differences among the TgFer structures before and after adding Cu^2+^ ions based on its biochemical characterization such as UV-vis and CD analyses. Nevertheless, there are still several other limitations to our study. On the one side, since the crystals of TgFer + Cu suffered severe cracking after long exposure times to the salt, we set the soaking time to 5 min. Although the crystals are performed with a short soak in mother liquor containing iron atom reagent, it seems highly likely that the soaking process can cause rough crystal surfaces and thus occur some low-resolution X-ray diffraction data and limited structure information. In this work, the crystal structures of TgFer + Cu and TgFer + CuFe had been solved by X-ray diffraction analysis at 2.3 and 3.9 Å resolution, respectively. Obviously, a low-resolution diffraction data not only present a challenge to structure determination, but also hamper interpretation of mechanistic details (Su et al., 2010). Moreover, it is indispensable for this research to provide the accurate assessment of oxidation kinetics via using stopped-flow experiments on the millisecond time scale. On the other side, our experiments only explore the protein structures more under crystallization conditions than under physiological conditions. Notably, small-angle X-ray scattering (SAXS) is useful for structural characterization of macromolecules in solution, which is radically different from crystal structure determination using X-ray crystallography (Korasick and Tanner, 2018). Therefore, further researches are necessary on the re-optimization of crystal growth and post-crystallization processing to improve the diffraction resolution limit of our crystals. Furthermore, complementary methods such as SAXS should be considered as a power tool for determining the in-solution structural properties of proteins.

## Conclusion

This study was performed to determine the crystal structural characteristics of TgFer, TgFer + Cu, TgFer + Fe, and TgFer + CuFe at resolutions of 1.78, 2.30, 1.85, and 3.90 Å, respectively. These structures exhibited a typically cage-like spherical shell composed of 24 identical subunits, in which the highly conserved organization of both the ferroxidase center and the 3-fold channel suggested the common function of directing iron into the cavity for Fe^2+^ ion oxidation. However, the structural and biochemical analyses indicated that the 4-fold channel of TgFer could be serviced as potential binding sites of metal ions. Structural comparisons revealed that although Cu^2+^ ion can compete with Fe^2+^ ion to bind to the structure of TgFer, Cu^2+^ binding to the 3-fold channel as well as ferroxidase site can potentially block Fe^2+^ binding, inhibiting the ferroxidase activity of TgFer. Further study will shed light on the role played by Cu^2+^ ion binding in strengthening the integrity and stability of the TgFer cage and for catalytic promotion by competing iron ions.

## Data Availability

The datasets presented in this study can be found in online repositories. The names of the repository/repositories and accession number(s) can be found below: http://www.wwpdb.org/, 6L55 http://www.wwpdb.org/, 6KZY http://www.wwpdb.org/, 6L56 http://www.wwpdb.org/, 6L58.
