# Supplementary material for: Structural Insights Into the Effects of Interactions With Iron and Copper Ions on Ferritin From the Blood Clam Tegillarca granosa
Source: Front Mol Biosci. 2022 Mar 11;9:800008. doi: 10.3389/fmolb.2022.800008 (PMC8961696; doi:10.3389/fmolb.2022.800008)
Supplement: Supplementary file 1 [file DataSheet2.PDF]

Table S1 Oligonucleotide primers used for site directed mutagenesis.

Ferritin variant	Oligonucleotide primers (5' to 3')
D129A/E132A	Forward: ATGCGCAGATGATGGCTTTCCTGGCAGGTGAATACCTGAA
	Reverse: TTCAGGTATTCACCTGCCAGGAAAGCCATCATCTGCGCAT
E168A	Forward: CATGTACGATAAAGCAACCATGAGCAGCTAA
	Reverse: TTAGCTGCTCATGGTTGCTTTATCGTACATG

Figure Legends

Fig. S1 Coordination environment for Fe and Cu ions at the ferroxidase site. (A) Coordination environment for Fe^{2+} ion in the TgFer crystal structure. **(B)** Coordination environment for Cu^{2+} ion in the TgFer+Cu crystal structure. **(C)** Coordination environment for Fe^{2+} ion in the TgFer+Fe crystal structure. **(D)** Coordination environment for Cu^{2+} ion in the TgFer+CuFe crystal structure. The model was superimposed on the 2Fo-Fc electron density map, contoured at 5.0 σ (blue mesh). The green balls represent water molecules.

Fig. S2 Ferroxidase sites and 3-fold channels of TgFer+Cu and TgFer+CuFe. (A) Coordination environment for Cu^{2+} ion at the ferroxidase site in the crystal structure of TgFer+Cu. **(B)** Coordination environment for Cu^{2+} ion at the ferroxidase site in the crystal structure of TgFer+CuFe. **(C)** Coordination environment for Cu^{2+} ion at the 3-fold channel in the crystal structure of TgFer+Cu. **(D)** Coordination environment for Cu^{2+} ion at the 3-fold channel in the crystal structure of Tgfer+CuFe. The model was superimposed on the anomalous-difference Fourier map contoured at 5.0 σ (blue wire).

Fig. S3 Coordination environment for Fe^{2+} and Cu^{2+} ions in the 3-fold channel. (A) Inner view of the structure of the TgFer crystal at the 3-fold channel. **(B)** Inner view of the structure of TgFer+Cu crystal at the 3-fold channel. **(C)** Inner view of the structure of the TgFer+Fe crystal at the 3-fold channel. **(D)** Inner view of the structure for the TgFer+CuFe crystal at the 3-fold channel. The model was superimposed on the 2Fo-Fc electron density map contoured at 5.0 σ (blue wire). The distance values are indicated as the mean ± SEM (n = 24 subunits). The green balls represent water molecules.

Fig. S4 Inner view of the 4-fold channel in the TgFer+Fe crystal structure. The model was superimposed on the 2Fo-Fc electron density map contoured at 5.0 σ (blue wire). The yellow dotted lines indicate the bonds between the Fe atoms (orange balls) and adjacent oxygen atoms of Glu168 residue.

Fig. S5 catalytic activities of TgFer and TgFer+Cu within the initial 100 s at 310 nm. (A–F) The Fe^{2+} ion/ferritin molar ratio ranged from 200 to 1200. The TgFer and TgFer+Cu samples without Fe^{2+} ion were used as blanks. For each sample, the average values of three replicates are shown.

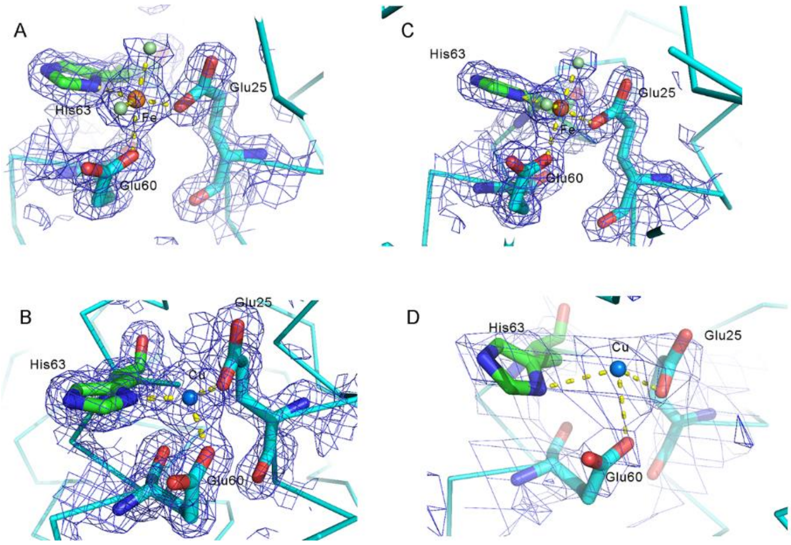
A
His63
Fe
Glu25
Glu60
C
His63
Glu25
Fe
Glu60
B
His63
Cu
Glu25
Glu60
D
His63
Cu
Glu25
Glu60

Fig. S1

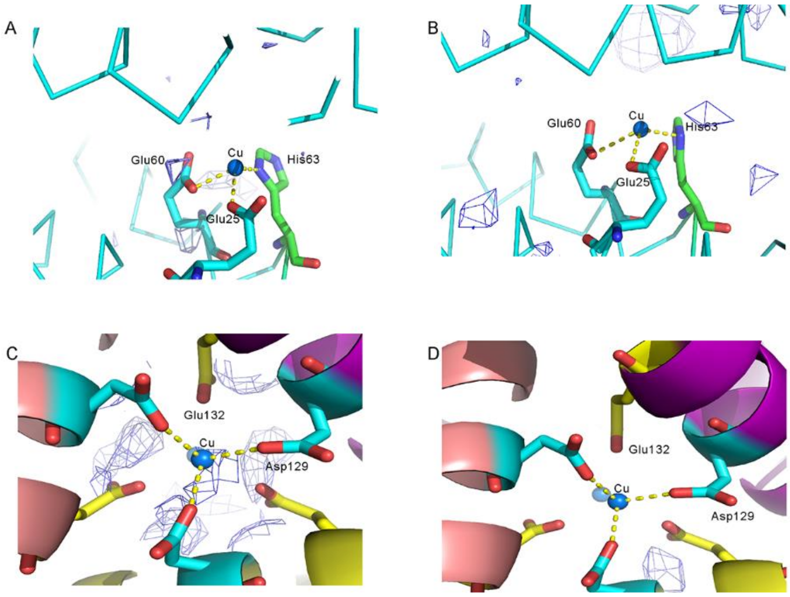
A
Glu60
Cu
His63
Glu25
B
Glu60
Cu
His63
Glu25
C
Glu132
Cu
Asp129
D
Glu132
Cu
Asp129

Fig. S2

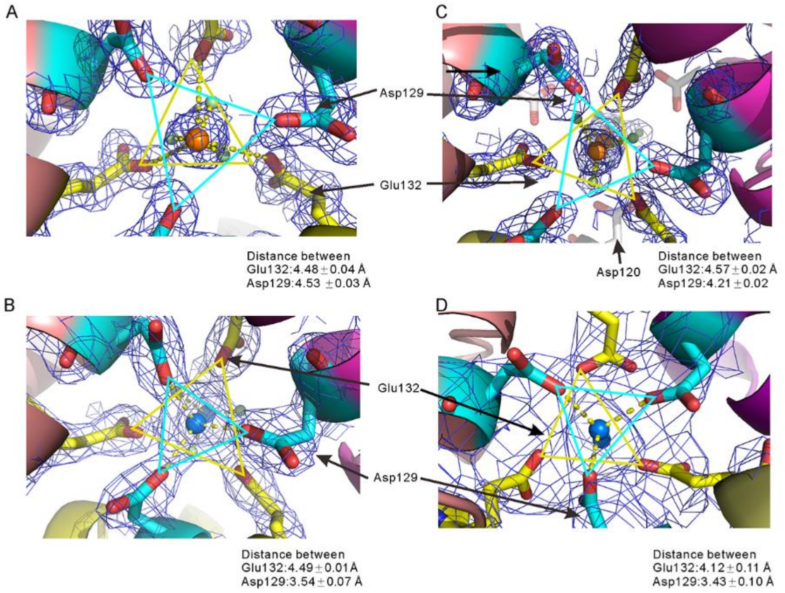
A
Asp129
Glu132
Distance between
Glu132:4.48±0.04 Å
Asp129:4.53 ±0.03 Å
C
Asp120
Distance between
Glu132:4.57±0.02 Å
Asp129:4.21±0.02
B
Glu132
Asp129
Distance between
Glu132:4.49±0.01 Å
Asp129:3.54±0.07 Å
D
Distance between
Glu132:4.12±0.11 Å
Asp129:3.43±0.10 Å

Fig. S3

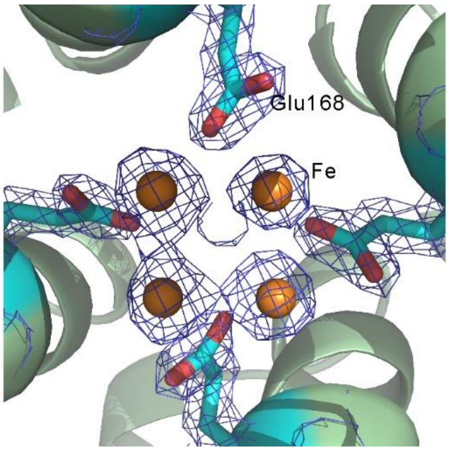
Glu168
Fe

Fig. S4

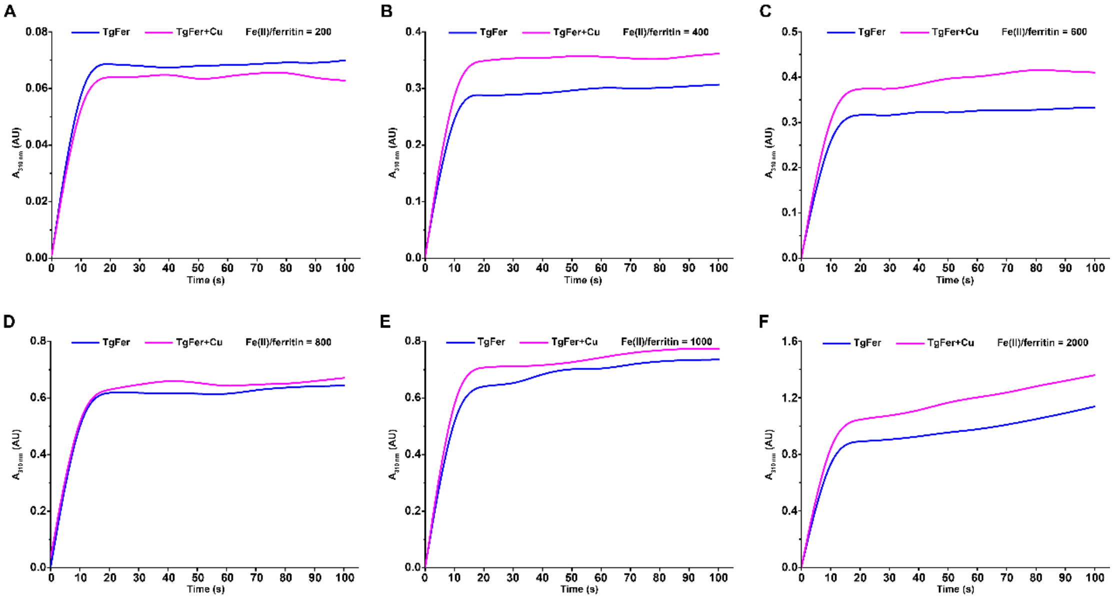
A
TgFer
TgFer+Cu
Fe(II)/ferritin = 200
B
Fe(II)/ferritin = 400
C
Fe(II)/ferritin = 600
D
Fe(II)/ferritin = 800
E
Fe(II)/ferritin = 1000
F
Fe(II)/ferritin = 2000
Time (s)

Fig. S5
