# Supplementary figures and images for: Structural Insights Into the Effects of Interactions With Iron and Copper Ions on Ferritin From the Blood Clam Tegillarca granosa
Source: Front Mol Biosci. 2022 Mar 11;9:800008. doi: 10.3389/fmolb.2022.800008 (PMC8961696; doi:10.3389/fmolb.2022.800008)

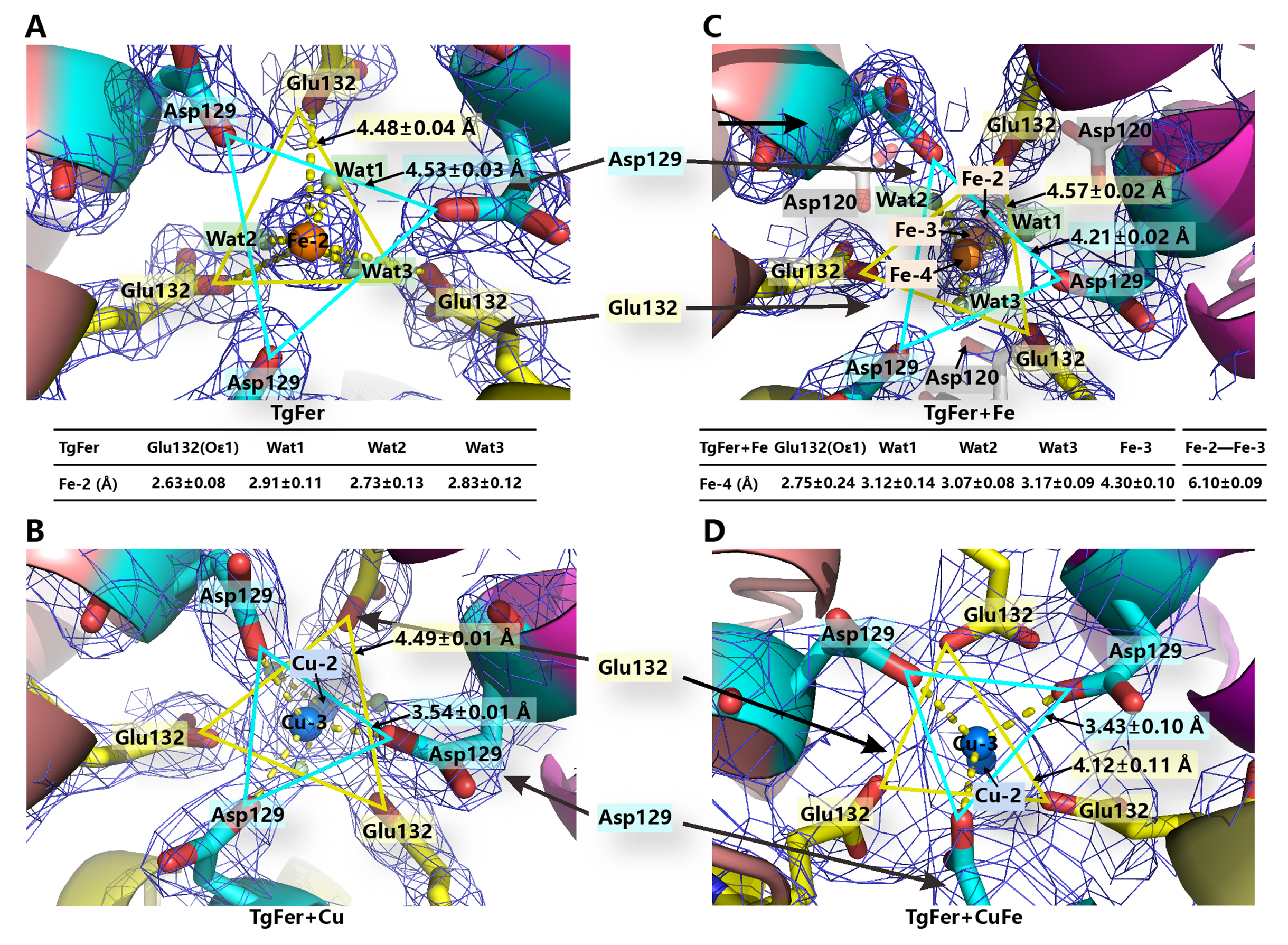

Supplement: Supplementary file 2 [file Image3.TIF]

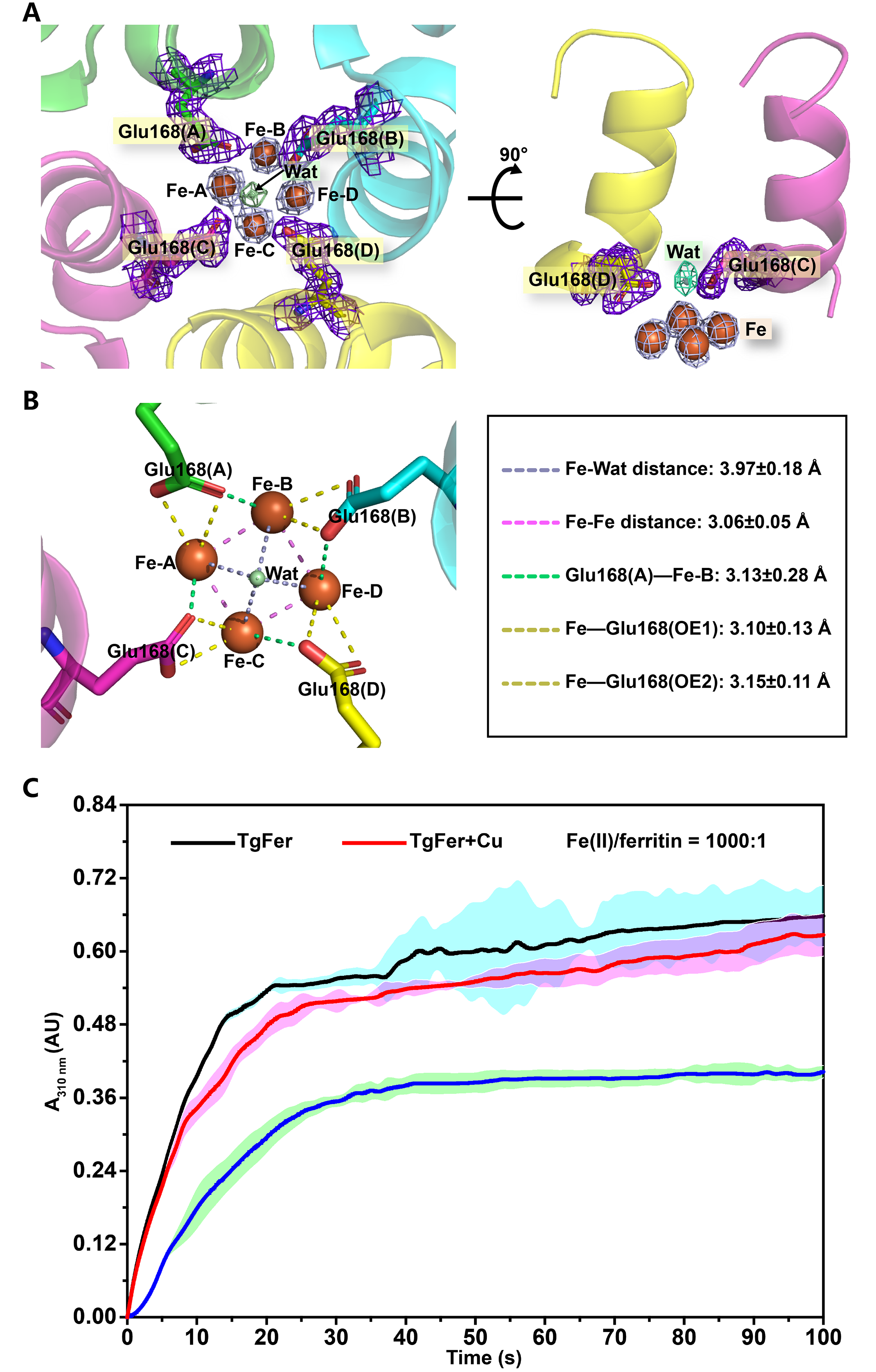

Supplement: Supplementary file 3 [file Image4.TIF]

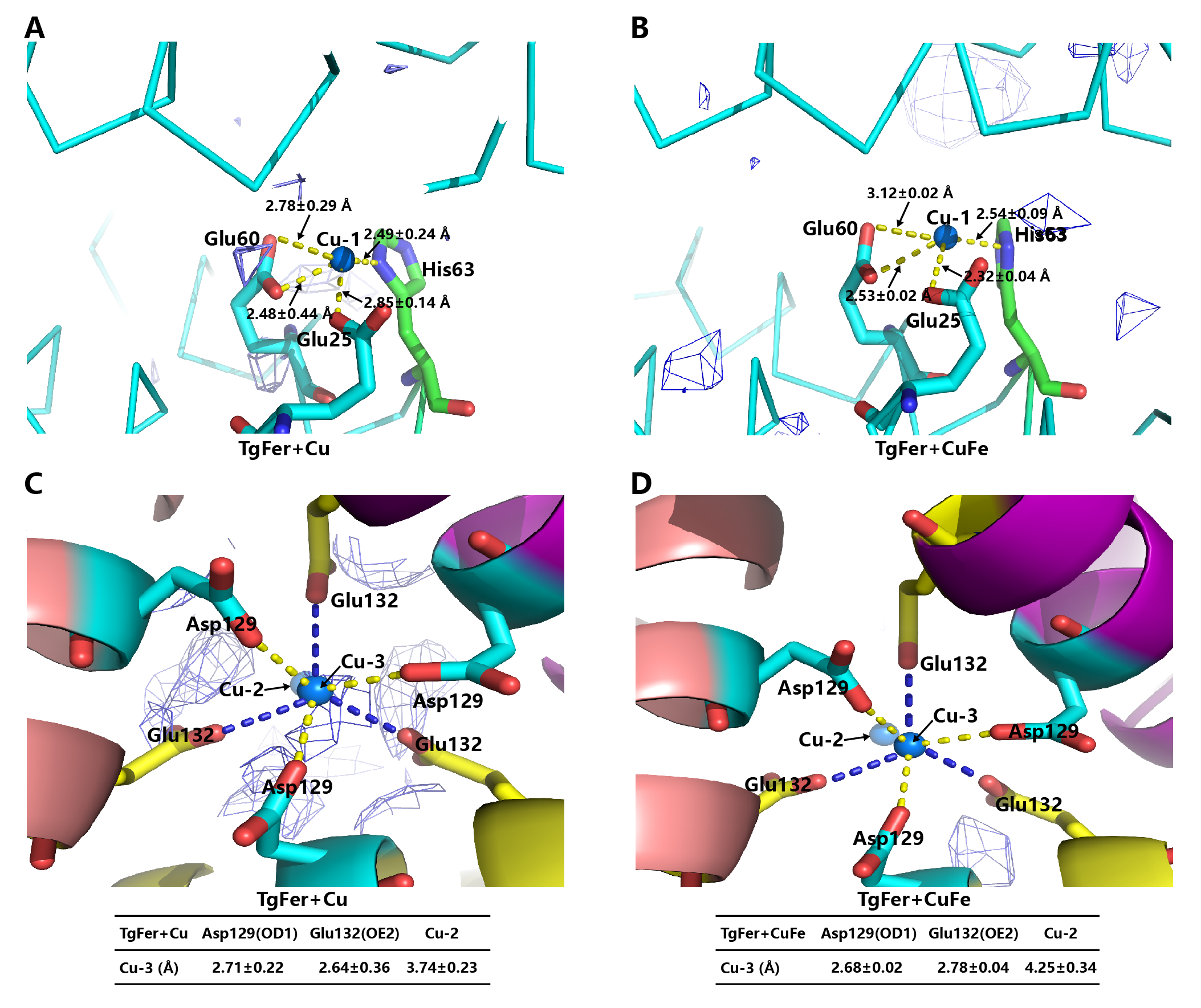

Supplement: Supplementary file 4 [file Image2.TIF]

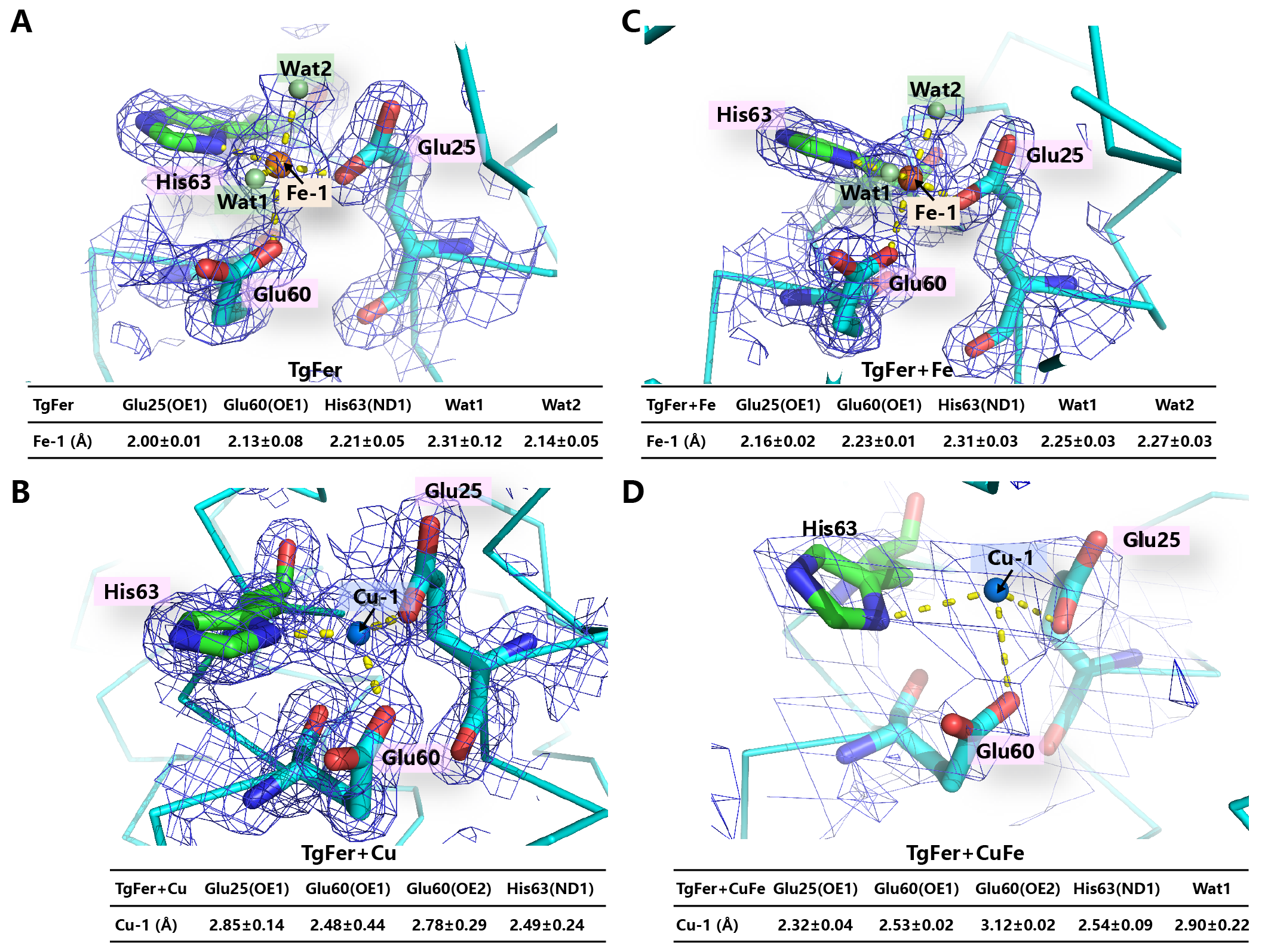

Supplement: Supplementary file 5 [file Image1.TIF]
