# Supplementary material for: Structural Insights Into the Effects of Interactions With Iron and Copper Ions on Ferritin From the Blood Clam Tegillarca granosa
Source: Front Mol Biosci. 2022 Mar 11;9:800008. doi: 10.3389/fmolb.2022.800008 (PMC8961696; doi:10.3389/fmolb.2022.800008)
Supplement: Supplementary file 7 [file DataSheet1.pdf]

Supplementary Material

Structural insights into the effects of interactions with iron and copper ions on ferritin from the blood clam *Tegillarca granosa*

Tinghong Ming[1,2]†, Qinqin Jiang[1,3]†, Chunheng Huo[1,2], Yan Wu[1,3], Hengshang Huan[1,3], Chang Su[4], Xiaoting Qiu[3], Chenyang Lu[1,2], Jun Zhou[1,2], Ye Li[1,2], Jiaojiao Han[1,2], Zhen Zhang[1,2] and Xiurong Su[1,2]*

†These authors contributed equally to this work.

*** Correspondence:** Xiurong Su: suxiurong_public@163.com

Supplementary materials legends

Table S1 Oligonucleotide primers used for site directed mutagenesis.

Table S2 The percentage contents of secondary structure of TgFer, TgFer+Cu, TgFer+Fe and TgFer+CuFe.

Figure S1 Coordination environment and metal-ligand distances for Fe and Cu ions at the ferroxidase site. (A) Coordination environment for Fe^{2+} ion in the TgFer crystal structure. **(B)** Coordination environment for Cu^{2+} ion in the TgFer+Cu crystal structure. **(C)** Coordination environment for Fe^{2+} ion in the TgFer+Fe crystal structure. **(D)** Coordination environment for Cu^{2+} ion in the TgFer+CuFe crystal structure. The model was superimposed on the 2*Fo-Fc* electron density map, contoured at 5.0 σ (blue mesh). The distance values are indicated as the mean ± SEM (n = 24 subunits). The green balls represent water molecules.

Figure S2 Ferroxidase sites and 3-fold channels representation as well as metal-ligand distances in TgFer+Cu and TgFer+CuFe. (A) Coordination environment for Cu^{2+} ion at the ferroxidase site in the crystal structure of TgFer+Cu. **(B)** Coordination environment for Cu^{2+} ion at the ferroxidase site in the crystal structure of TgFer+CuFe. **(C)** Coordination environment for Cu^{2+} ion at the 3-fold channel

in the crystal structure of TgFer+Cu. **(D)** Coordination environment for Cu^{2+} ion at the 3-fold channel in the crystal structure of Tgfer+CuFe. The model was superimposed on the anomalous-difference Fourier map contoured at 5.0 σ (blue wire). The distance values are indicated as the mean ± SEM (n = 24 subunits).

Figure S3 Coordination environment and metal-ligand distances for Fe^{2+} and Cu^{2+} ions in the 3-fold channel. (A) Inner view of the structure of the TgFer crystal at the 3-fold channel. **(B)** Inner view of the structure of TgFer+Cu crystal at the 3-fold channel. **(C)** Inner view of the structure of the TgFer+Fe crystal at the 3-fold channel. **(D)** Inner view of the structure for the TgFer+CuFe crystal at the 3-fold channel. The model was superimposed on the 2*Fo-Fc* electron density map contoured at 5.0 σ (blue wire). The distance values are indicated as the mean ± SEM (n = 24 subunits). The green balls represent water molecules.

Figure S4 Close views of 4-fold axis channel structure of the 4-fold channel in the TgFer+Fe crystal structure. The selected 2*Fo-Fc* electron density maps at 1.0 σ are shown in greencyan and lightblue, respectively. Iron ions are shown as orange spheres of arbitrary radius, and the 2*Fo-Fc* electron density map contoured at 1.5 σ (purple wire). The coordinating water molecules are represented as green small spheres. **(A)** Views of iron ion coordination inside the 4-fold pores in TgFer+Fe. **(B)** The yellow and green dotted lines indicate the bonds between the Fe atoms and adjacent oxygen atoms of Glu168 residues. The metal coordination bonds both Fe-Fe and Fe-Wat are shown in pink and lightblue stick representations, respectively. The distance values are indicated as the mean ± SEM. **(C)** Iron oxidation kinetics of the D129A/E132A and E168A mutants with Fe^{2+} ion/ferritin molar ratio of 1000:1 within the initial 100 s at 310 nm. The background oxidation of iron was used as the blank. After subtraction the absorbance value of the blank, the average values of three replicates for protein samples are calculated. Solid lines represent the average (n=3) of technical replicates, and the shaded areas represent the standard deviation from the mean.
